# The Dutch COVID-19 Notification App: Lessons Learned From a Mixed Methods Evaluation Among End Users and Contact-Tracing Employees

**DOI:** 10.2196/38904

**Published:** 2022-11-04

**Authors:** Joris Elmar van Gend, Jan Willem Jaap Roderick van 't Klooster, Catherine Adriana Wilhelmina Bolman, Julia Elisabeth Wilhelmina Cornelia van Gemert-Pijnen

**Affiliations:** 1 The BMS Lab Faculty of Behavioural, Management and Social Sciences University of Twente Enschede Netherlands; 2 Department of Psychology Open University of the Netherlands Heerlen Netherlands; 3 Section of Psychology Health & Technology, Department of Technology, Human and Institutional Behavior Faculty of Behavioural, Management and Social Sciences University of Twente Enschede Netherlands

**Keywords:** eHealth, contact tracing, digital contact tracing, contact-tracing apps, COVID-19, adherence, public health, mobile health, topic analysis, health service, user experience, eHealth intervention, mobile phone

## Abstract

**Background:**

The Dutch CoronaMelder (CM) app is the official Dutch contact-tracing app (CTA). It has been used to contain the spread of the SARS-CoV-2 in the Netherlands. It allows its users and those of connected apps to anonymously exchange warnings about potentially high-risk contacts with individuals infected with the SARS-CoV-2.

**Objective:**

The goal of this mixed methods study is to understand the use of CTA in the pandemic and its integration into the Municipal Health Services (MHS) efforts of containment through contact tracing. Moreover, the study aims to investigate both the motivations and user experience–related factors concerning adherence to quarantine and isolation measures.

**Methods:**

A topic analysis of 56 emails and a web-based survey of 1937 adults from the Netherlands, combined with a series of 48 in-depth interviews with end users of the app and 14 employees of the Dutch MHS involved in contact tracing, were conducted. Mirroring sessions were held (n=2) with representatives from the development (n=2) and communication teams (n=2) responsible for the creation and implementation of the CM app.

**Results:**

Topic analysis and interviews identified procedural and technical issues in the use of the CTA. Procedural issues included the lack of training of MHS employees in the use of CTAs. Technical issues identified for the end users included the inability to send notifications without phone contact with the MHS, unwarranted notifications, and nightly notifications. Together, these issues undermined confidence in and satisfaction with the app’s use. The interviews offered a deeper understanding of the various factors at play and their effects on users; for example, the mixed experiences of the app’s users, the end user’s own fears, and uncertainties concerning the SARS-CoV-2; problematic infrastructure at the time of the app’s implementation on the side of the health services; the effects of the society-wide efforts in containment of the SARS-CoV-2 on the CM app’s perception, resulting in further doubts concerning the app’s effectiveness among MHS workers and citizens; and problems with adherence to behavioral measures propagated by the app because of the lack of confidence in the app and uncertainty concerning the execution of the behavioral measures. All findings were evaluated with the app’s creators and have since contributed to improvements.

**Conclusions:**

Although most participants perceived the app positively, procedural and technical issues identified in this study limited satisfaction and confidence in the CM app and affected its adoption and long-term use. Moreover, these same issues negatively affected the CM app’s effectiveness in improving compliance with behavioral measures aimed at reducing the spread of the SARS-CoV-2. This study offers lessons learned for future eHealth interventions in pandemics. Lessons that can aid in more effective design, implementation, and communication for more effective and readily adoptable eHealth applications.

## Introduction

### Background

One of the measures that numerous countries have implemented to mitigate the spread of the SARS-CoV-2 is the use of contact-tracing apps (CTAs) [1], defined as “software that can be installed on a user’s personal device, such as a smartphone to notify the user when they come into contact with a person or persons infected with SARS-CoV-2” [2]. Studies have revealed epidemiological impact; large numbers of cases of COVID-19 infection would be averted by this digital contact tracing [3] mainly through the potential increase in speed of the contact-tracing process. However, estimates show that more than half of the population must simultaneously use the app for it to be effective [4].

### The Dutch CoronaMelder App

The Dutch government has developed a CTA called CoronaMelder (CM) based on the Google Apple Exposure Notification system jointly developed by Apple and Google [5]. The app was implemented and made available to the public in October 2020. The Dutch CM app aimed to (1) relieve the Municipal Health Service (MHS) in contact tracing, (2) identify and trace high-risk contacts earlier and faster than manual contact tracing of the MHS, and (3) inform the users who have been in the proximity of an infected app user about the measures that were strongly advised to be taken (eg, undergo a polymerase chain reaction test, quarantine, not to have visitors at home, and social distancing) [6] to avoid (further) potential spread of the virus. The basic mechanism of the app is to classify the prolonged (for at least 15 minutes) proximity of one app user to a second app user who has tested positive for COVID-19 infection as a high-risk contact. The app will then anonymously send the first user a notification, alerting them about their high-risk contact in the app. This notification recommends that the user undergo a test at the MHS and quarantine until the test result is received. If the user receives a positive test result, the app offers the user the ability to notify other app users with whom they in turn have been in contact. In the Dutch situation, the MHS is a linking pin in most cases, that is, in the case of a positive test, the user is flagged as infected in the MHS’ systems and contacted to start the (manual) contact-tracing process. The MHS official (health official) subsequently asks the end user to read a code generated by the app, which the health official then adds to their system to be validated as a code for an infected individual. Next, the end user can choose to send a notification to warn others through the app [7]. Alternatively, since October 2021, the user can validate the code themselves through a 2-step verification–protected MHS website that provides them with positive test results [8].

### State of CTAs and Research

This study is the second qualitative study on the Dutch CM app and part of a larger effort to evaluate the effectiveness of the app as a tool during the COVID-19 pandemic. Earlier research [9] focused on the usability and user-centeredness of the app during its initial release. Changes were made to the app because of this first study. CTAs have also been introduced in other countries. Although algorithmic improvements were made to increase the accuracy of CTAs in some countries (including the Netherlands), most countries relied on the Google Apple Exposure Notification reference framework. The implemented possibilities to contact the authorities after a positive test differ by country, and adoption rates vary by country as well. Before the spread of the omicron variant of the SARS-CoV-2, epidemiological studies in the United Kingdom have found that CTAs are effective when adopted by the public [3]. A German study [10] found that a higher level of education led to improved adoption of pandemic apps. In Belgium, a survey found a large majority of nonusers This refusal of use was largely due to privacy concerns and ambivalence about the app’s utility [11]. In Greece, an in-depth additional contact-tracing training study found that contact-tracing efforts work best in a systematic and coordinated manner, and systematic and organized training of contact-tracing workers can greatly increase its effectiveness [12]. Thus, it is important to consider not only the app but also the privacy concerns and embedding in the public health infrastructure.

### The Dutch CM App and Contact-Tracing Process Over Time

The Dutch app is continuously being monitored and evaluated by the Dutch Ministry of Health, Welfare and Sport for its adoption and effectiveness. Studies have revealed that approximately one-third of the Dutch population downloaded the app approximately 1 year after its launch [13]. The lowest adoption has been found among people with lower education levels, lower monthly incomes, and those aged >80 years [6]. The CM app has proven to trace and alert a substantially higher number of contacts than MHSs; 77% of those who were notified by the app had not been contacted by the MHS [14] in their manual contact-tracing process. The results are less positive regarding behavioral outcomes, such as adherence to measures designed to prevent the spread of the virus that are provided in the app. Only 45% of the users stayed at home after notification, and only 41% of the users applied for a polymerase chain reaction test. Little is known about the causes of behavioral outcomes. For example, no insights have been gained at that point into the user friendliness of the app and its comprehensibility [9]. This changed after the initial usability study of the CM app by Bente et al [8]. The same goes for the extent to which the behavioral measures (presented as recommendations to users, such as self-isolation) the app provides are perceived as both clear and unambiguous and to what degree the app’s notifications might trigger potentially unintended and undesired effects (eg, anger or panic). Finally, it was not known why users might have chosen not to follow these measures and how the app’s integration with the work processes of the MHS might have affected the app’s effectiveness. However, these insights are essential to determine whether and why the app is used correctly and does or does not enforce the proposed behavioral measures. Eventually, such information is vital for improving the CTA and its integration into the contact-tracing process of the MHS and ultimately, to support its sustainability and scalability. The latter will remain of paramount importance as the COVID-19 pandemic has been shown to be persistent with periodically fluctuating levels of infections and social control measures. Digital contact tracing remains an important part of the strategy to suppress the spread of the SARS-CoV-2.

### Challenges From Privacy by Design

Because the app was designed in accordance with the *privacy by design* principle, it is complicated to gather the aforementioned information quantitatively via the app. Moreover, options for data collection that do exist are limited to numbers, such as the number of notifications sent in a period, which do not shed light on underlying motives or causes for behavior, such as adherence to behavioral measures or a lack thereof. This study builds upon and follows up on the results from the evaluation of the CM app and is part of the continuous evaluation of the CM CTA [8,13]. The integration of the CM app in contact tracing of the MHS will also be explored. As such, this study takes 2 viewpoints into account: those of end users and employees of the Dutch MHS involved in the contact-tracing process. Moreover, the study uses a mirroring approach on the development and communication teams involved in the creation and implementation of the app to gain insights into the context of the findings and the feasibility of implementing improvements that are advised. The research questions are as follows: (1) What hinders, deters, or motivates end users (citizens) in adopting the app and their adherence to the app’s instructions and advice (behavioral measures)? (2) How is the app implemented in the work processes of the Dutch MHS’ contact-tracing teams? (3) How does CTA use affect adherence to isolation and quarantine measures?

## Methods

### Overview

To gain the necessary insights into the first and third research questions (use of the app, adherence of CM app users, and user friendliness), 3 methods were applied. First, a topic analysis (n=56) to get a first understanding of the pain points encountered when using the app was conducted using emails sent by users of the app. Second, a short web-based survey (n=1802) was conducted to gain insights into the adoption of the app and the degree to which app notifications were received and sent and to gather the contact information of positively tested individuals who used the app and who would be invited to participate in in-depth interviews. Third, semistructured interviews (n=48) were conducted to gain deep insight into adherence to behavioral measures and perceptions of the app. The second research question was studied through semistructured interviews with contact-tracing employees of the MHS. The findings were then discussed with the teams of the Dutch Ministry of Health, Welfare and Sport and the MHS responsible for building and maintaining the app and communicating about the app. The design and procedures of this study were evaluated by the responsible ethics committee (Multimedia Appendix 1) of the University of Twente’s BMS faculty.

### Topic Analysis on Emails Sent by Users of the App

The topic analysis was based on 56 participants who reacted by email on a call in a regional newspaper (November 11, 2020, Twentsche Courant Tubantia) [14], which requested that they share their experiences with the CM app as part of scientific research. Out of 63 mails, 56 (89%) met the inclusion criteria. Participants were included if they had installed and activated the CM app, received a notification, and possibly shared their app’s (MHS) key with MHS employees to warn other CM app users. The 56 participants selected formed a varied sample from across the Netherlands. The sentiment in their emails was analyzed using manual coding. This means that 2 coders would independently read each email and determine whether the sentiment of the email was generally positive, negative, or neutral. Similarly, a list of possible topics was identified in the first round of analysis and then coded by 2 independent coders in the final analysis. Conflicting assessments of coders were resolved through a third reviewer’s evaluation or the email in question.

### Web-Based Survey

A web-based survey was sent between December 1, 2020, and December 21, 2020, via a Dutch panel service called “Panelclix.” The main goal of this survey was to recruit potential participants for the interviews. The selection criteria were age (>18 years) and education; the aim was to have a sample group in which 70% of the participants had a lower and middle level of education. Participants were asked 9 questions concerning the use of the CM app, whether they had received a notification or, in case of a corona infection, whether they had shared their CM app MHS authentication key with the MHS. Panel members who received notification were asked to participate in the interviews (on the web or by phone). The full set of questions from the survey is included in Multimedia Appendix 2 and is in the Dutch language.

### Interviews

#### Interviews With CM App Users

A total of 48 web-based interviews were conducted between December 1, 2020, and January 21, 2021, with participants who used the CM app, received a notification (38/48, 79%) or had tested positive for COVID-19 infection, after which they had or had not shared the MHS key with the MHS (8/48, 16%). Some participants (3/48, 6%) fulfilled both the criteria. The targeted sample consisted of individuals from the general population, with a focus on hard-to-reach groups, such as individuals with low education levels, limited reading or digital skills, a migration background, and adults over the age of 65 years. Multiple recruitment channels were used, such as the media (outreach through the newspapers), the CM app helpdesk, schools, companies, a library, a football club, a health care organization, and organizations that support individuals with a low level of literacy such as “Pharos” and “Stichting ABC.” Participants were asked questions about factors that influenced their adherence behavior regarding COVID-19–related behavioral measures, their underlying motivations for their adherence (or lack thereof), and the limiting or facilitating factors they experienced within the CM app during their use. Moreover, questions were asked to explore their experience with the work processes of the MHS if the interviewed individual was contacted by an MHS contact-tracing employee. Important factors (eg, technical problems) identified during the topic analysis and the survey were used in the design of the questions for the interview with contact-tracing employees. The full list of interview questions and subjects can be found in Multimedia Appendices 3 and 4 and are in the Dutch language.

#### Interviews With Contact-Tracing Employees

To explore the embedment of the CM app in the contact-tracing process at the MHS, 11 semistructured web-based interviews were conducted with contact-tracing employees of the Dutch MHS. The interviews were conducted between January 28, 2021, and February 10, 2021. The participants were recruited through a network of researchers, and an open invitation was posted at 2 MHS locations. Participants were asked about the process of contact tracing, the manner in which they communicated with the index (person tested positive for COVID-19), the extent to which the CM app was embedded in this process, the limitations and difficulties they experienced in their work, and how these could be reduced. Moreover, the results of both the topic analysis and interviews with CM app users served as a basis for the questions in the interviews with MHS employees. Issues identified by users in the MHS processes and uncertainties about the MHS practices were integrated into the interview questions.

All interview participants, both app users and MHS employees, received a gift card after the interview in exchange for their time investment. The overall results were communicated and mirrored by the designers and creators of the CM app and the communication team of the Dutch Ministry of Health, Welfare and Sport responsible for the campaigns concerning the CM app to gain additional insights and aid in the app’s further development and implementation campaign. The app and the workflow and training of the MHS employees were adapted because of this mirroring (see Discussion).

#### Analysis of Both Interview Series

Semistructured interviews were conducted in accordance with the list of subjects and questions. The interviews were recorded, transcribed verbatim, and coded by 2 independent coders. The subjects mentioned and statements made by the participant related to the questions, and subjects on the list were coded and then added to the count of the appropriate code on the coding scheme or added as a new code. Because of this method, the sum of the frequencies of individual codes under a code group (subject) does not always correspond to the total number of participants. There are 2 reasons for this finding. First, frequencies represent the counts of codes in statements made by the participant. Not all participants made statements that qualified for a code for each code group, resulting in a lower sum of frequencies than the total number of participants. Second, some participants may have made statements to which multiple codes of the same code group applied, resulting in a higher sum of frequencies. The coding scheme consisted of a limited set of codes created beforehand based on the characteristics of the app (thematic coding), procedures from the contact-tracing teams, and information and adherence recommendations that were provided in the app and input from both topic analyses, which were supplemented with codes identified while analyzing the interviews. Coders returned to previously analyzed transcripts to verify that statements to which newly created codes would apply were not missed. Conflicts between coders were resolved through discussion and a third coder that could weigh in.

### Feedback by Stakeholders

The findings of the 3 substudies were presented to and discussed with the teams responsible for the realization and implementation of the app (MHS, design team, and communication team). In total, 2 sessions were held for approximately an hour with 2 representatives from the development team and 2 from the communication team of the Dutch Ministry of Health, Welfare and Sport. This allowed them to apply the findings to the redesign of the CM and implementation of the CM in the work processes of the MHS and to tailor the communication campaign. Moreover, it provided this study with the opportunity to incorporate the challenges faced from a policy, technical and communication perspective in the realization, implementation, and use of the CM app and its processes. Thus, this study incorporated insights from various stakeholders.

### Ethical Considerations

Participants of all 3 substudies were informed about their participation beforehand and could withdraw their participation at any point of time. In the case of topic analysis, this was done by explicitly mentioning that the emails sent to researchers would be analyzed and experiences distilled and anonymously used for scientific research. In the case of both the survey and interviews, an informed consent procedure was followed, as outlined in the application to the University of Twente’s Ethical Review Board (approval number 201323) for the Behavioral Sciences (Multimedia Appendix 1). The purpose of this evaluation was to independently assess and address (potential) ethical concerns regarding the study or its compliance with applicable legislation.

## Results

The results of the topic analysis are presented first, followed by the interviews with the CM app users and the results of the interviews with the contact-tracing employees. Selected quotes from participants were translated literally and used to illustrate the results. The original untranslated quotes are provided in Multimedia Appendix 5.

### Topic Analysis

#### Overview

In total, 56 people sent usable emails after the publication of a call (outreach) to share their experiences with the CM app. Of the 56 participants, 22 (39%) were male, 30 (54%) were female, and of the remaining 4 (7%) participants, sex could not be determined with certainty from the contents of their email. Participants were aged between 20 and 80 (mean 55, SD 15.0388) years. A relatively large group (10/56, 18%) was ≥65 years. The contents of the emails were unprompted and thus reflected the experiences that were most focal to the participants.

All but one of the participants (55/56, 98%) indicated that they had received a notification from the CM app warning them of high-risk contact with an individual who tested positive for COVID-19 infection. Initial responses to the notification were mostly emotional and strongly negative and manifested in the form of anger, fear, and disbelief. A common theme in the emails was the uncertainty participants felt regarding the contact that triggered the notification and the meaning and implications of the notification (eg, How high is the risk? When was the contact exactly? What to do now?). Moreover, during the early phases of the app’s use, the phone’s operating system would periodically send notifications that confused the participants. The notifications would inform the user that they had spent a week without having any high-risk contact. These notifications were often mistaken for notifications intended to warn about past high-risk contacts and caused dissatisfaction and anger:

On Monday 31 Augustus, I read on my CoronaMelder that I had been close for more than 15 minutes to someone who reportedly was infected. It was a big shock! That, according to me, wasn’t possible! But, anyway, I called the phone number that was provided. I got the advice to go in quarantine and to call my general practitioner or the MHS if symptoms developed. I called my friends and family! The Monday afterwards I received another notification from the CM app. I had not been in contact with anyone infected according to the app. I didn’t understand this at all!

MAIL0116

Notifications regarding high-risk contacts were often perceived as arriving too late. Most commonly (17/56, 30%), participants indicated that they had received a notification within 5 days after their high-risk contact. However, a significant group was indicated to have received the notification between 5 and 10 days (11/56, 20%) or more than 10 days after the high-risk contact:

After reading your article in the newspaper, I would like you to know that I have removed the app [CM] from my phone and this is why: On 2 November I received a message that I had been in contact for more than 15 minutes with someone that has corona and that I had to stay inside until 30 October.

MAIL0072

#### Response to Notifications

Participants (7/56, 13%%) expressed severe doubts about the validity of the notification they received. For example, participants knew that they had not left the house on the day of the supposedly high-risk contact. Others could identify the source of the notification as their neighbor they had not seen in person. They then deduced that the Bluetooth signal must have traveled through the walls of their homes. A different subsection consisting of 7 (13%) participants indicated that their first course of action after receiving a notification was to verify its validity because of doubts. Participants indicated that these occurrences undermined their confidence in the validity of the notifications and the CM app, which resulted in a more complicated decision-making process regarding whether they should go into quarantine or isolation:

My experience is that the phones of the neighbours and myself had been in contact. ... It can happen that people get a notification [from CM] that is incorrect, but better this than the other way around.

MAIL0074

To relieve anxiety and doubts created by the receipt of notifications, participants, for example, chose to contact the MHS or their general practitioner. Their aim was to remove some of the uncertainty and gain insights into the meaning and implications of the notification and actions to be taken:

After yet another sleepless night I concluded that I still did not experience any symptoms. To get rid of all the brooding, I made an appointment with the branch of [a commercial test provider] in [the city of] Hengelo.

MAIL0209

When participants were able to secure a COVID-19 test and received a positive test result, the MHS had to contact them to start the contact-tracing process. However, participants indicated that this contact moment had not occurred, and others indicated that when this did happen, the MHS did not ask about their use and thus the possibility of sending a notification through the CM app.

#### Overall Sentiment

Of the emails analyzed, 34 were coded as containing a primarily negative sentiment regarding the CM app. By contrast, 12 emails were coded as having neutral and 10 as positive sentiments.

### Web-Based Survey

An invitation to fill in the web-based survey was sent to 7489 individuals in the Netherlands who were selected based on their age (>18 years) and education level (mainly those with medium or lower education level were targeted). In total, 1802 (24.06%) people participated in the survey (Table 1). A total of 54.61% (984/1802) of the participants had never installed the CM app at that point in time (autumn 2020). A large group of 692 (38.40%) participants had the app installed at that moment, and a smaller group of 126 (6.99%) participants indicated that they had installed the app at some time but had removed it since then. Participants that had never installed the app were from here on out excluded from the analysis. Out of the participants that had the app installed at one point (n=818), most participants (728/818, 89%) had not received a notification about high-risk contact from the app, while 55 (6.7%) participants received 1 notification and 26 (3.2%) received multiple notifications. A small group of participants (37/818, 4.5%) tested positive at some point before completing the survey. At the time of the survey, most participants (755/818, 92.2%) had not tested positive for COVID-19. Some participants (5/818, 0.6%) did not wish to divulge this information or provided no answer (9/818, 1.1%). Of the 37 participants who tested positive, 22 (59%) decided to share the app’s key with the MHS to anonymously notify their contacts, while 15 (41%) did not. Of the 22 participants who decided to share the key with the MHS, 3 (14%) decided not to warn their contacts through the CM app. Participants who had installed the app at some point, received a notification or had tested positive (86/818, 11%) were asked to provide demographic data and participate in the follow-up interviews. Of the 86 participants, most were male (45/86, 52%). The participants had a mean age of 44.8 (SD 17.2) years. A total of 40.7% (35/86) of the participants had a low education level, 38.3% (33/86) had a medium education level, and 20.9% (18/86) had a high education level.

**Table 1:** Overview of results from the web-based survey (N=1802).

Subject and code			Count, n (%)
**Installation of app (n=1802)**			
	Never	984 (54.61)	
	Currently installed	692 (38.4)	
	Installed at some point	126 (6.99)	
**Received a notification (n=818)**			
	None	728 (88.9)	
	One notification	55 (6.7)	
	Multiple notifications	26 (3.1)	
	No answer	9 (1.1)	
**Tested positive (n=818)**			
	Yes	37 (4.5)	
	No	755 (92.2)	
	Other or do not want to share	5 (0.6)	
	No answer	21 (2.6)	
**Shared key with Municipal Health Service (n=37)**			
	Yes	22 (59)	
	No	15 (41)	

### The CM App User Interview Results

The results of the app user interviews were grouped and presented for each subject. Each subject is briefly introduced and a table with the results can be found at the end of each section.

#### Sample Characteristics

In total, 48 CM app users were interviewed. They were recruited using various methods. A large group was recruited through outreach efforts by various organizations, such as local newspapers (33/48, 69%), low-literacy support organizations (2/48, 4%), and local professional football club supporters (4/48, 8%). Furthermore, a web-based panel (2/48, 4%, derived from the aforementioned survey), the Dutch CM app helpdesk (2/48, 4%), a local trade school (1/48, 2%), and other means (5/48, 10%) provided the remaining participants. Of the participants who were interviewed, a little more than half were identified as female (27/48, 56%) and the rest as male (21/48, 44%). Participants were aged between 21 and 80 years with a mean age of 51 (SD 16.2) years. The level of education among the participants was not representative of the Dutch population and skewed toward a high level of education (34/48, 71%) as opposed to middle (7/48, 15%) or low (7/48, 15%). The recordings of 3 (6%) interviews were not usable because of technical problems. Thus, these participants were excluded from the analysis, leaving 45 interviews for this analysis.

#### Installation of the App

Table 2 displays the division of the installation date across the sample. among the CM app users that were interviewed, there were 3 major points in time at which the app could be installed. First, when the app was in a local pilot-testing stage (22/49, 46%). Second, when it initially became available to the public in September 2020 (22/45, 49%). Third, after December 1, 2020 (1/45, 2%), at which point in time a major update was released for the app and the associated MHS processes [13]. The major update included the ability for CM to exchange codes with CTAs from other European countries, changes to texts in the app, and the update to MHS procedures, which allowed people to schedule a COVID-19 test if they indicated not to experience any COVID-19–related symptoms. A small group (5/45, 11%) decided to uninstall the app between its initial release and the interview period, and a slightly larger group (7/45, 16%) predicted that they would uninstall the app in the near future:

My daughter in law owns a hair salon in [city], which is now closed as well. She had 5 people working for her and had the CoronaMelder. She removed it. She indicated that the app drove her crazy.

CME008, male, 71 years, high level of education

**Table 2:** Overview of results on installation and deletion of CoronaMelder app (N=45).

Subject and code		Value, n (%)
**Date of installation**		
	During pilot	22 (46)
	After pilot and before December 1, 2020	22 (46)
	After December 1, 2020	1 (2)
**Removed app**		
	Before December 1, 2020	5 (11)
	After December 1, 2020	0 (0)
	Will remove app in future	7 (16)

#### Receiving a Notification

**Figure 1:**
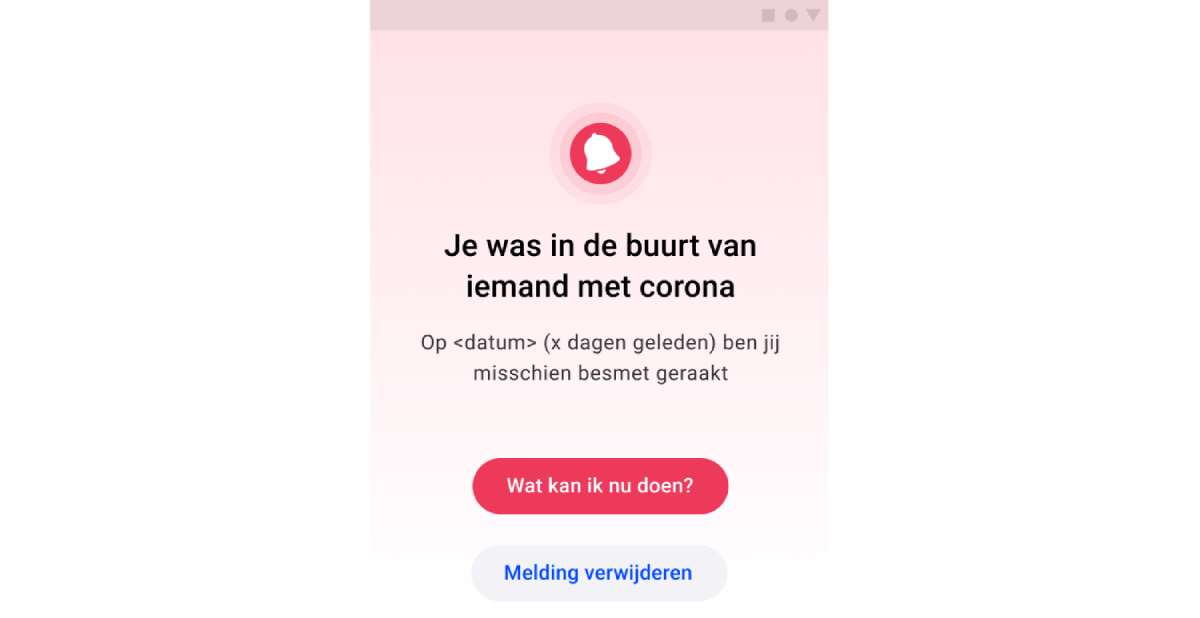
Screenshot from the main menu of the app when the user was warned about a high-risk contact containing 4 main elements: (1) Title reading “You have been in the vicinity of someone with corona”. (2) Description reading “You were at an increased risk of infection on <date> (x days ago).”. (3) Button reading “What can I do now?”. (4) Button reading “Delete notification”.

Users of the Dutch CM app received a notification if the app determined that they were at an increased or high risk of being infected with the SARS-CoV-2. This is the case when the app registered that the user was near (closer than 1.5 meters) another app user for a prolonged (>15 minutes) period who had tested positive for COVID-19 infection through an official Dutch test center. The notification becomes visible in the notification bar of the user’s phone and in the main menu of the CM app (Figure 1). The results regarding this subject are found in Table 3. Most participants (30/45, 67%) received 1 notification, some (8/45, 18%) received multiple notifications, and a smaller group (7/45, 16%) did not receive a notification.

**Table 3:** Overview of results on receipt of notifications (N=45).

Subject and code			Value, n (%)
**Number of notifications received**			
	None	7 (16)	
	One notification	30 (67)	
	Multiple notification	8 (18)	
**Prevalence of symptoms at the time of notification (n=38)**			
	None	30 (79)	
	Some	8 (21)	
**Prevalence of symptoms after receiving the notification (n=38)**			
	None developed	26 (68)	
	Some did develop	4 (11)	
	Already had symptoms	8 (21)	
**Actions with notification (n=38)**			
	Removed notification	10 (26)	
	Saved notification	12 (32)	
**First response about notification (n=38)**			
	Negative (eg, angry or upset)	4 (11)	
	Fear or anxiety	23 (61)	
	Disbelief or amazement	14 (37)	
**Beliefs about validity of notification (n=38)**			
	Doubtful or invalid	26 (68)	
	Valid	12 (32)	
**First action after receiving notification (n=38)**			
	Contacted friends or family	18 (47)	
	Contacted work	4 (11)	
	Investigated the validity and origin of notification	29 (76)	
	Removed the CoronaMelder app	3 (8)	
	Contacted a medical professional (eg, Municipal Health Service or general physician)	18 (47)	
	Requested test	9 (24)	
	Remained watchful of symptoms developing	4 (11)	
**Response to advice given in notification (n=38)**			
	Followed fully or partially	23 (61)	
	Not followed	9 (24)	
	Advice was read	6 (16)	

Among those that had received a notification (n=38), the most common initial responses to receiving a notification were shock (23/38, 61%) and anger and outrage (4/38, 11%). The latter response was largely due to notification being perceived as arriving very late by the participants (9/38, 24%). This was days after the participants had already received news of high-risk contact through other channels or thought that they had already endangered others:

I had already received a WhatsApp message in the morning from someone whom I had exercised with that they were positive. Therefore, I already knew that I had to take action. I had a sore throat ache, but I doubted a bit. Then the CM notification came and that felt more serious.

CME0146, female, 58 years, high level of education

I was scared to death. You receive a notification that you were in contact with someone who was infected with corona on the 17th of October. That made me think: Where was I on that Saturday? What was I doing? I don’t go to the market or store. I started thinking and I really thought hard about it. It gave me sleepless nights and I never figured out where I had been and who I had been in contact with.

CME0017, male, 65 years, middle level of education

Participants reported problems in the process of receiving notifications, such as receiving notifications late at night that woke them up or scared them. Others attempted to contact the MHS and were unable to do so, owing to busy lines or contact information that was misunderstood. Most participants (26/38, 68%) doubted whether the notification was accurate or justified, while only 12 (32%) participants were convinced of its correctness:

I know that I was supposed to have had a contact on 29 October and that I received the notification on the Sunday (8 November) afterwards. This, on the moment when my quarantine period had elapsed, I received the notification.

CME0109, female, 45 years, middle level of education

Because that same day I also called the MHS, but it was not possible to get through. So, then I called again the next day.

CME0104, male, 59 years, high level of education

Participants undertook various (and often multiple) actions after receiving a notification and often mentioned multiple actions. These actions varied from trying to verify the notification’s correctness (29/38, 76%), contacting a branch of the health services or a general practitioner (19/38, 50%), contacting friends and family (18/38, 47%), contacting work (4/38, 11%), being watchful of symptoms (4/38, 11%), and uninstalling the app (3/38, 8%). Overall, participants indicated that they were uncertain or completely unaware of what to do:

But I did call all those people in the meantime. And those people were not afraid either, they were happy that I had called them and that was it.

CME0008, male, 71 years, high level of education

What do you do: You are going to think about where I had been and what I had done that day. And you can’t figure it out properly and you thus become irritated.

CME0125, female, 67 years, low level of education

#### Perception of and Complying With Isolation and Quarantine Advice

The Dutch CM app provides information on whether one should go into quarantine after receiving a notification or should isolate themselves when receiving a positive COVID-19 test result. The advice was shown to all participants (n=45) in the study. Most of the advice given was understood, and the participants indicated that they had complied with them in general (23/45, 51%). Table 4 provides the values per advice. Participants, however, indicated that they did not understand the advice to keep distance from coinhabitants of the same house; in-depth interview questions revealed that they misunderstood the advice (7/45, 16%). The participants did not follow up on this advice (15/45, 33%):

Yes, but my husband and I live with just the two of us, so that’s not applicable. ... My husband and I lie next to each other in 1 bed, so that is not going to work out.

CME0006, female, 62 years, high level of education

It was my daughter’s birthday when my son was infected. My daughter then sat downstairs with some friends and my son was upstairs. We didn’t say, at that point in time: No one is allowed into the house.

CME0031, female, 50 years, high level of education

**Table 4:** Overview of results on response, understanding, and adherence to advice provided in the CoronaMelder app (N=45).

Subject and code			Value, n (%)
**Advice to stay at home**			
	Was recognized	35 (78)	
	Was executable	24 (53)	
	Did follow	28 (62)	
	Did not follow	8 (18)	
**Advice to have others do grocery shopping**			
	Was recognized	28 (62)	
	Was executable	24 (53)	
	Did follow	24 (53)	
	Did not follow	3 (7)	
**Advice to maintain distance from room- and housemates**			
	Was recognized	27 (60)	
	Not recognized or interpreted wrongly	7 (16)	
	Was executable	17 (38)	
	Not executable	5 (11)	
	Did follow	13 (29)	
	Did not follow	15 (33)	
**Advice to have not visitors**			
	Was recognized	32 (71)	
	Was executable	20 (44)	
	Did follow	23 (51)	
	Did not follow	2 (4)	
**Advice to seek medical help when symptoms worsen**			
	Was recognized	20 (44)	
	Was executable	13 (29)	
	Did follow	19 (42)	
**Advice to get tested for COVID-19 infection when symptoms present themselves**			
	Was recognized	25 (56)	
	Was executable	12 (27)	
	Did follow	16 (36)	
	Did not follow	2 (4)	

#### Acquiring a COVID-19 Test and the Test Result

A key mechanism of the process behind the Dutch CM app is the acquisition of an official COVID-19 test after receiving a notification. A total of 24 people requested a test (Table 5), of which 13 (54%) did so at a branch of the Dutch MHS, of which 4 (31%) participants did so at a location run by general practitioners. The other 9 (69%) participants went for a COVID-19 test at another type of location, run either commercially or by their employers. Most participants (8/11, 73%) who did not go to a testing location run by the Dutch MHSs indicated that the reason for this was the perceived unavailability and slowness of the Dutch health services in scheduling tests and communicating results. Most participants received their test results between 24 and 48 hours (7/24, 29%) or within 24 hours (6/24, 25%) after the test. Results regarding the receival of test results are found in Table 6. Some participants (5/24, 21%) received their test results after 48 hours. Most participants (13/24, 54%) acquired their results through a protected government website, indicating that this was the easiest way. Participants who tested positive (13/24, 54%) were all called by the health services, which served as the start of the contact-tracing process. A total of 14 participants reported having gone in isolation sometime after a positive test, 11 (79%) of whom reported having received a notification from the app before going to the test:

At the MHS, the problem at that point in time was that their capacity was too limited for the number of requests they received. It was known at that time to be the case. That was quite a big hassle and irritator. However, it was easily arranged at a commercial test centre, so that was a good experience.

CME0227, male, 35 years, high level of education

So, then I was tested, but my symptoms weren’t severe enough. I also couldn’t get a test immediately. Well, that was what the girl on the phone said, “You don’t have symptoms.” However, I then told her that I had been in contact with someone that had been infected and that I’d like to be tested to be sure. I said that I might have experienced mild symptoms in the throat, but I don’t think that I would have had myself tested with those symptoms under normal circumstances. I did it to make the situation more severe so I could get tested.

SME 0042, female, 62 years, high level of education

He was tested on Tuesday evening and was called on Thursday afternoon that his results were negative. In the meantime, we had drawn the conclusion that his test result would be negative, but it caused him a lot of stress in the meantime. Particularly because he knew the test result was available. But that they then waited for 1.5 days to call him, was pretty frustrating.

CME0028, female, 36 years, middle level of education

**Table 5:** Overview of results on requesting COVID-19 tests (n=45).

Subject and code			Value, n (%)
**COVID-19 test requested**			
	Test requested	24 (53)	
**Method or channel of request (n=24)**			
	Employer	1 (4)	
	Coronatest website (MHS^a^-run website)	6 (25)	
	Phone	7 (29)	
	General practitioner	1 (4)	
	Unknown or uncertain	9 (38)	
**Reason for use of method or channel of request (n=24)**			
	Speed or ease of web-based channel	6 (25)	
	Ease of starting a call from the CoronaMelder app	2 (8)	
	Higher availability	3 (13)	
	Priority or employer arranged	4 (17)	
	Unknown or other	7 (29)	
**Type of testing facility used (n=24)**			
	MHS	13 (54)	
	Facility run by general practitioners	4 (17)	
	Commercial	3 (13)	
	Unknown	4 (17)	
**Reason for using a non-MHS test** **(n=11)**			
	Speed or capacity of the MHS test insufficient	8 (72)	
**Time between receipt of notification and execution of test (n=24)**			
	<24 hours	6 (25)	
	<48 hours	2 (8)	
	>48 hours	1 (4)	
	Not applicable (no notification)	2 (8)	
	Unclear or uncertain	11 (46)	
**Isolation (n=24)**			
	Went into isolation after test	14 (58)	
	In isolation after notification and test	11 (46)	

^a^MHS: Municipal Health Service.

**Table 6:** Overview of the results on receival of test results (n=24).

Subject and code			Value, n (%)
**Channel through which test result was received**			
	Phone	4 (17)	
	SMS text messaging or email	2 (8)	
	On the web	14 (58)	
**Test result**			
	Negative	10 (42)	
	Positive	13 (54)	
	Unknown	1 (4)	
**Time between COVID-19 test and test result**			
	<24 hours	5 (21)	
	24-48 hours	7 (29)	
	>48 hours	5 (21)	
	Unknown	4 (17)	
**Actions after test result**			
	Removed app	4 (17)	
	Informed acquaintances or work	1 (4)	
	Searched for support (eg, Municipal Health Service)	3 (13)	

#### Sending a Notification After a Positive Test Result

**Figure 2:**
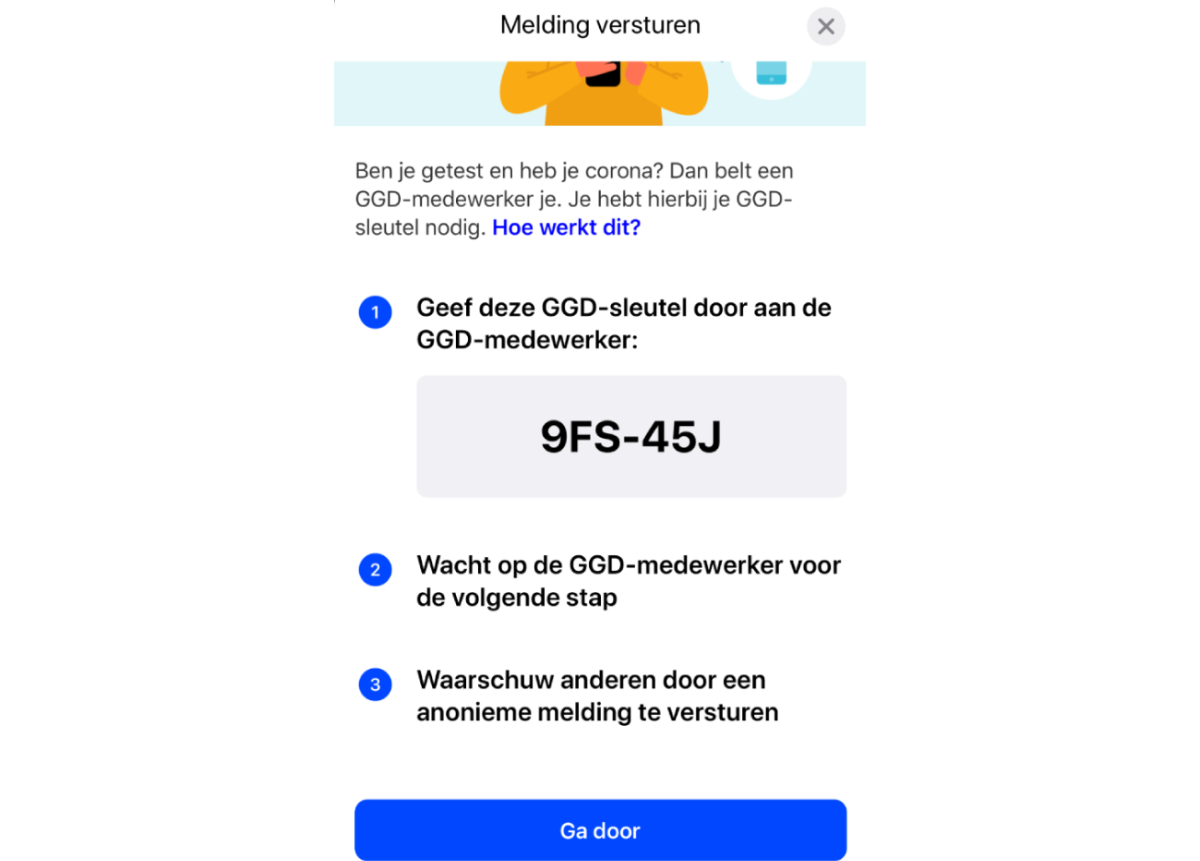
Screenshot showing the screen where CM app users would start the process of sharing their anonymously gathered keys. The screen shows the three steps needed before a notification is send and other users are warned. The translation of the steps is as follows: "1 Give this MHS key to the MHS employee", "2 Wait on the MHS employee for the next step" and "3 Warn others by sending an anonymous notification.".

The Dutch CM app offered the option of warning others to whom a user had been in close proximity after they had received a positive test result for COVID-19 infection themselves. See Table 7 for the actions taken by participants regarding sharing their result and sending a notification. In total, 13 participants received positive test results. Participants were divided with regard to the action to be taken after receiving a positive test result. Some participants removed the app (4/13, 31%); sought help from branches of health services or general practitioners (3/13, 23%); or immediately started contacting relatives, friends, and colleagues (1/13, 7%). The participants could send notifications to their contacts. They first had to share their app’s key (“the MHS key”) with the health services while on the phone, which 11 (85%) participants did. They then had to send the notification through the app (Figure 2). In total, 5 out of 11 (46%) participants succeeded in doing so and thus completed the notification-sending process. In several cases (5/11, 45%), the option to send a notification was either not offered or unavailable. Overall, 2 out of 13 (15%) participants reported a positive experience in sending notifications:

Because of this, you also start doubting the effectiveness of other things, like the CM app. I had assumed that the hospital would have shared the positive test result. ... But in my case, the hospital hadn’t communicated the positive test result with the MHS.

CME0426, male, 69 years, education unknown

And that was the next day, so on Monday the 19th I had a person on the phone about contact tracing. They asked whether I had been in contact with people. I told them that I hadn’t been in contact with many people, I had been in contact with the physical therapist. They asked me if I could inform them myself and wanted to hang up. I then said that I had the CM app and asked if I could do something with it. They ten told me to provide the code, so they could report it. That I did. However, if I hadn’t told them ... Then they would have just asked me to inform others and nothing else.

CME0179, male, 70 years, high level of education

No, because I assumed that the MHS would do that. ... Because the MHS asked me for the key and it wasn’t clear to me that I had to finish the rest of the procedure.

CME0179, male, 70 years, high level of education

The screenshot in Figure 2 has been translated as follows: (1) title: “sending a notification.” (2) description: “Have you been tested, and do you have COVID-19 infection? Then an MHS employee will call you. The employee will help you warn others who have been in your vicinity. You will need the MHS key for this.” (3) blue hyperlinked text: “How does this work?” The 3-step plan needed for notification sending and reading, “1. Pass this MHS key through to the employee: A56-34F. 2. Wait on the MHS employee for the next step. 3. Warn others by sending an anonymous notification.” (4) A button saying “Continue,” which allows users to continue the process and afterward confirm the sharing of their anonymously gathered codes through a pop-up from the operating system.

**Table 7:** Overview of results on sharing of the Municipal Health Service (MHS) key with the MHS (n=13).

Subject and code		Value, n (%)
**Shared MHS key with MHS**		
	Did share	11 (85)
	Did not share	0 (0)
	Did not mention or uncertain	2 (15)
**Experience with sharing the key**		
	Positive	9 (69)
	Negative	4 (31)
**Send notification**		
	Did send	5 (38)
	Did not send	5 (38)
	Did not mention or uncertain	3 (23)
**Experience with sending notification (n=11)**		
	Positive	2 (18)
	Negative	2 (18)
**Reason for not sending notification**		
	MHS will take care of this	5 (45)

#### Overall Attitude Toward the App and the Improvements in and Strengths of the App

Most participants (37/45, 82%), as shown in Table 8, perceived the app positively overall:

But if you get a notification and you stay inside, then you cannot infect anyone else and then it won’t spread as much. So yes, absolutely.

CME0006, female, 62 years, high level of education

However, a significant group (13/45, 29%) had doubts (also among those that perceived the app positively) about its effectiveness or was decidedly negatively inclined (3/45, 7%) toward the CM app. Participants experienced much uncertainty in case they received a notification and indicated (5/45, 11%) that they wanted clarification on when and why notifications were sent and the level of certainty that the high-risk contact could be determined:

My brother-in-law, for example, received a notification as well, but afterwards it turned out that he wasn’t infected. You have to be close to someone for 15 minutes, but he says he wasn’t. So that makes you doubt a bit, whether that was good. Is it [CM app] functioning 100%?

CME0006, female, 62 years, high level of education

Others (4/45, 9%) noted that the time between the contact for which they received a notification and the actual moment of receiving the notification was too long (eg, sometimes more than 4 days later). Respondents indicated that this undermined their confidence in the CM app’s efficacy. The notification-sending process itself was unclear to some extent (4/45, 9%). Some wanted more functionality or capability (5/45, 11%) of the app and better and more graphically oriented content (5/45, 11%). Finally, some expressed that CM app use (5/45, 11%) or adherence to the recommendations provided by the app after a notification (2/45, 4%) needed to be communicated and stimulated among the public. Participants classified the app’s ease of use (18/45, 40%) and user friendliness and the recommendations provided by the app as its strengths.

**Table 8:** Overview of results on overall attitude, strengths, and points for improvements of the CoronaMelder (CM) app (n=45).

Subject and code		Value, n (%)
**Overall attitude toward the CM app**		
	Positive	37 (82)
	Had doubts on effectiveness	13 (29)
	Negative	3 (7)
**Potential improvements to the CM app**		
	Clarify sending of notification	2 (4)
	Reason and process of receiving notification is unclear	5 (11)
	Time between high-risk contact and notification is too long	4 (9)
	Increase functionality	5 (11)
	Stimulate app use	5 (11)
	Stimulate adherence to advice	2 (4)
	Add more graphics in the CM app	3 (7)
**Strengths of the CM app**		
	Clear advice	7 (16)
	Easy to use or clear	18 (40)
	User-friendly and good layout	9 (20)
	Anonymity or privacy	1 (2)

### Results of the Interviews With Contact-Tracing Employees

In total, 14 interviews were conducted with the employees of the Dutch MHS. Of these participants, 13 (93%) worked as contact-tracing employees who were responsible for contacting individuals with a positive COVID-19 test result and finding their source of infection and those that they might have infected. A participant worked as a medical adviser for the policy branch of Dutch MHS and was involved in the writing and creation of protocols and procedures for contact-tracing employees. The results are presented in text without tables, as the relatively low number of participants did not warrant the use of tables. Most participants that were active in contact tracing had worked in their roles for between 3 and 6 months (9/13, 69%). Others had worked as contact-tracing employees for <3 months (2/13, 15%). Most participants (8/13, 61%) indicated to have worked for the health services of a single region, while a single participant indicated that they had worked for more than one region. All participants that were active in contact tracing indicated that they had received some form of training in contact tracing. Most participants (10/13, 77%) indicated that their training involved some form of training on using the Dutch CM app. Of those, 7 (70%) indicated that they had been sufficiently trained in both their primary contact-tracing process and the use of the Dutch CM app. The most often cited reason for those that reported to have received insufficient training (n=6) is that they lacked practical examples and ways to handle them (4/6, 67%) and that they would have liked more training in conversations (2/6, 33%) or parts of the CM app (4/6, 67%):

And the training is very theoretically good, and you think that this is how it will go. And then you start working, and you notice that it doesn’t go like they said in the instructions. So you do learn useful skills, but in the end those conversations are different. Mainly because they are less scary. The instruction makes it seem like everyone is constantly angry and is going to threaten your life, but I never experienced this.

CME 1008, male, 26 years, high level of education

What we are missing, and we pointed that out yesterday, is that we need more depth. It all remains a bit superficial.

CME1007, female, 28 years, high level of education

Overall, 9 out of 14 (64%) interviewed participants had a generally favorable attitude toward the Dutch CM app, and 4 (28%) had a neutral or negative attitude. Only half of the participants (7/14, 50%) were app users.

Most participants (11/14, 79%) indicated that the app was only helpful in some cases, was never helpful, or that they were unsure about the app’s effectiveness. The most often cited reason for this (4/11, 36%) was that participants experienced that only a small portion of their contact-tracing indexes using the app:

I don’t use the [CM] app myself because I have the idea that it gets used too little to be truly useful. I think, that if I were to be infected, that I wouldn’t be warned through the app.

CME1008, male, 26 years, high level of education

### CM App in the Contact-Tracing Employee’s Work

#### Procedures and Workload

Once on the phone with their contact-tracing subject (“index”), the participants that worked in contact tracing (n=13) had to walk through extensive checklists and procedures that took >2 hours on average per subject, according to most contact-tracing employees (7/13, 54%). Only 3 (23%) contact-tracing employees reported spending <2 hours on average per index:

Lately, I have conversations of an hour and the time needed for administration is at least an hour as well. Sometimes even longer, depending on where someone has been. Thus, it is very hard for me to make an estimation of it [time spent per index]. Often, it takes longer than expected.

CME1009, female, 25 years, high level of education

Participants indicated that they were often (6/13, 46%) able to contact their indexes between 24 and 48 hours after the positive test result. Moreover, 2 (15%) participants reported that they often had to contact indexes well after 48 hours had passed since the positive test result was known. For most indexes, this was their first opportunity to learn about test results.

A majority of participants (5/13, 38%) reported working according to the procedures provided by the National Office of the Health Service or a local variation in these procedures (2/13, 15%). A total of 3 (13%) participants reported that they found the procedures and their status to be unclear. Only 2 (15%) participants indicated that there was a clear set of instructions to use the CM app. A majority participants (10/13, 77%) reported that they had inquired about whether the CM app was installed by the contact-tracing indexes who they contacted. However, inquiries were of limited scope, as only 4 (30%) participants reported asking whether the contact-tracing indexes had received a notification from the app themselves. Moreover, the registration of these data about the app was also reportedly limited. Only 2 (15%) participants indicated that they had asked for the first day of the onset of the symptoms, and only 8 (62%) inquired whether the app was currently in use by the contact-tracing subject:

The way in which I do my contact tracing, I do not explicitly ask for it [use of CM app]. Maybe a link could be added that if you make an appointment and it is because of the CM app, that it automatically adds this in the system. When we get the test results, it then reads “This person is warned by the app.” That would be a way to make that clear. At this moment there are several ways into the test centres. You can call because you experience symptoms, you can also call because you were warned by the CM app or because you are in quarantine for 5 days. We currently do not see information about this in the system.

CME1000, male, 29 years, highly educated

Participants working in contact tracing reported that they had to register data about the CM app in a myriad of different systems, but most commonly in one of the following systems called the “HP zone” (10/13, 77%), “CoronIT” (7/13, 54%), or special checklists (4/13, 31%). They reported that they often had to register the same data in multiple systems, and hence, the overlap in reported counts. When asked to estimate the percentage of their contact-tracing indexes using the CM app, most reported <15% (5/13, 38%) or between 15% and 30% (4/13, 31%). Moreover, according to 4 (31%) participants, a data breach at the health services at a certain moment in the CM implementation period lowered the willingness of people to share the required code to send a notification.

Only 4 (31%) MHS contact tracing employees who were interviewed reported that they had no difficulties with the CM app. Participants reported providing information about the CM app to contact-tracing indexes only to a limited degree. A total of 8 (62%) participants reported that they emphasized the importance of the subject sharing the MHS key, of which 2 (25%) participants reported explaining the process behind it as well. Only 5 (38%) participants indicated that they had separately mentioned the importance of the subject pressing the button in their CM app to send the actual notification. Participants (3/13, 23%) reported that the indexes would change their willingness to share their CM app key after having the process explained to them. A total of 8 participants reported having encountered troubles with the key sharing and notification-sending process. Most often (3/8, 38%), it was owing to a (temporary) disruption of service; other times (2/8, 25%), the subject fell into a demographic (eg, high-school students) who at that time were not asked to share their key, and 2 (25%) participants reported having trouble completing the process itself:

The reason why they don’t share the keys is because they didn’t know that they had to provide information. Why they don’t know that, is something I don’t know.

CME1006, male, 44 years, high level of education

#### Improvements to the Contact-Tracing Process and CM App

The participants (n=14) were asked to describe the weaknesses of this system and some improvements. A total of 6 (43%) participants reported that the process was too slow. Some of these participants (3/6, 50%) thought that part of the process or workflow concerning the CM app could be shortened. They suggested achieving this by allowing users of the app to share their keys themselves and notify their contacts without needing contact with the MHS. Moreover, 5 (36%) participants would have liked to pay more attention to the CM app in the protocol for contact tracing. They indicated that they saw potential leads for their contact-tracing work by inquiring more deeply about CM app use; for example, on whether the index had decided to have themselves tested because of a notification. Moreover, 5 (36%) participants liked to see the app use promoted among the public.

### Results Mirroring the Approach

The results of the combined research methods were discussed during 2 meetings with both the team responsible for developing and implementing the app and the team responsible for the (mass media) communication about the CM app. The results were discussed chronologically and resulted in the following changes to the CM app.

First, both end users and contact-tracing employees pointed out that the need for the MHS to be on the phone with an index to send a notification through the CM app was a limitation of the app’s mechanisms. Delays were introduced into the MHS chain during periods when the number of infections was very high. Consequently, the delay between the initial infection and the moment of notification sending would increase to such a degree that it would hamper the CM app’s ability to send out the warning in a timely manner. An update in December 2021 made it possible for infected individuals to send their MHS code to the MHS through a website on which they could see their test results to speed up notification.

Second, early in the interviews, it became apparent that hospitals that administer their own COVID-19 tests did not share their results in such a way, with the MHS, that a CM app notification could be sent to the patient’s contacts. It was made part of the hospital protocols to participate in the CM app notification-sending process before January 2021.

Third, the mirroring sessions with the build and communication teams revealed that there was a lack of awareness concerning the importance of the total time between the first and second generations of potentially infected individuals going into quarantine. In other words, the time between individual A being infected and being notified about this and individual B, who had been in contact with A, receiving a notification from individual A about their own potential infection is a crucial time window. Stakeholders, such as build and communication teams, often used a narrower definition of this window, which excluded the part where individual B would be informed and quarantined. This resulted in an underestimation of the time window and a delay or prioritizing of changes that could shrink the time window. Both the communication and build teams embraced this broader definition, and measures were taken to reduce delays at each step of the process as a result.

Fourth, notifications sent during the night were perceived as annoying and scary. Participants would wake up from it and would not be able to go back to sleep. The app now takes the time of day into account and does not send notifications during the night.

Fifth, fears, misconceptions, and concerns identified to be prevalent among the CM app users were addressed on various information channels. The CM app’s in-app information was updated, processes clarified, and the information provided by the official Dutch (government) channels was amended.

## Discussion

### Overview

This study investigated the implementation, adoption, and use of the Dutch CM app using a mixed methods approach. The app provides a set of features that, in theory, can greatly enhance the capability to perform contact tracing to control the spread of an infectious disease as, for example, studies of this app [8,13] and international equivalents [15] have shown. Adoption of the app and adherence to the advice (behavioral measures) are, however, key elements in the app’s effectiveness This study identified issues within the app that undermine the adherence (use of the app) and implementation (adoption). This chapter answers the main research questions, makes comparisons to earlier work, discusses the strengths and weaknesses of the study, and provides a conclusion.

### Principal Findings

Findings are discussed using the 3 research questions posed in the Introduction.

#### Factors Affecting End-User Adherence and Adoption of the App

First, the elements of the app, such as the way notifications were structured, the time of day at which notifications would appear, and the seemingly high error rate of notifications as reported by a large majority of participants, caused distress and dissatisfaction and undermined user trust.

Second, because of a lack of understanding of the mechanisms behind the app on the side of the users and MHS personnel, users reported not starting or aborting the key sharing and notification-sending processes. Moreover, MHS personnel were not adequately trained and motivated to consistently explain and encourage the use of key sharing and notification-sending functionalities.

Third, the interval between an individual getting tested, receiving a positive result, sending out a notification, and their contact receiving the notification and quarantining was key to creating and maintaining confidence in the app’s effectiveness. Participants reported intervals longer than desired. Various technical and administrative actions have been suggested to reduce this interval and have since been implemented. This illustrates the importance of paying attention to the larger system and context in which the app exists. Participants indicated that they had lost (parts of) adherence to behavioral measures as a result.

Finally, the scope of the CM app was limited to an app that could warn potentially infected contacts. Users expected and indicated that they desired more of the app. A study by Blasimme et al [16] shows that adding new features may “be seen as one way to deliver more personal utility to app users, thus incentivizing participation.” Instead of adding features, the reopening of society was, for example, facilitated by a separate app “CoronaCheck,” and the needs around the psychological aspects of isolation were not met at all [17]. Thus, the opportunity to enhance the CM app was missed, which the German “CoronaWarn,” for example, used.

#### Implementation in the MHS Work Processes

The CM app is intended as a tool within the broader contact-tracing process in which its purpose is to speed up parts of this process, allow for earlier warning, and reduce the load on the MHS. However, this study found that it was underused. This was especially relevant in times when infections were high, and the manual contact-tracing process was less effective. Hence, the integration with the MHS systems and processes is important for its effective functioning. However, the CM app received too little importance within the MHS; training in using and understanding the value of the app was perceived as inadequate, the data provided by the app were scarcely used, and motivation among MHS employees in using the CM app and trust in the app’s effectiveness were lacking. Moreover, the overall MHS processes were perceived as fragmented and cumbersome by the employees, which further negatively affected the MHS employees’ motivation to include and use the CM app. As a result, the CM was not consistently part of the MHS process, and its potential strengths in allowing for faster and earlier contact tracing did not materialize in full. Here, an app such as the CM offers a unique value that was not used.

#### CTA Use and Adherence to Quarantine and Isolation

The COVID-19 pandemic presents the first case in the Netherlands, in which technologies such as the CM app were used as a society-wide intervention to increase adherence to behavioral measures. As a result, this study brought to light lessons on the mistakes and importance of integration between different interventions and communication campaigns from different organizations. First, the CM app itself provided coherent and consistent messaging on behavioral measures, and its users were motivated to follow this. Second, the effectiveness of the CM app was partly dependent on its inclusion in a wider narrative set through communication campaigns and health policies. Conflicting or changing narratives and changing and nontransparent policies contributed to a lowering of trust in the CM app as a tool and adherence to its advice. Moreover, the reverse was observed during the study, and the performance of the CM app affected the overall perception of those narratives and policies. Third, the mirroring and action research approach used in this study served as an effective method to achieve rapid change and helped stakeholders in the app improve their understanding of both the daily use and reality of the app’s users and the broader context in which they were the stakeholders themselves. Such changes can help increase adherence in situations such as during the COVID-19 pandemic, where uncertainty is high and change rapid. The effectiveness of the CM app in encouraging adherence behavior was limited, but its potential was great.

### Comparison With Prior Work and Strengths

This mixed methods study provided a broad and in-depth insight into the functioning of the CM app, adherence to the actions, and contact-tracing process of MHS. To our knowledge, this study is the first to provide such a broad and simultaneously deep view. Other studies have focused on singular aspects, such as usability [9], its epidemiological impact [14], and factors contributing to the adoption of similar apps [9,16]. This study provides both a deep and broad view of the app and the environment in which it exists.

A strength of this study is the “mirroring approach.” The results have been translated into concrete recommendations for optimization of the CM app for designers as well as for the national system for contact tracing under the MHS care. Recommendations were discussed within and between these teams and resulted in feasible and actionable changes that affected users’ adherence. For example, sharing the key has changed, which means that users can upload the key without contacting the MHS. The findings were also used to set up a novel communication campaign to improve the adoption of the CM. Thus, this study has proven to be a valuable contribution to better use of the CM and optimization of the contact-tracing process. To our knowledge, this is the first study to have attempted such an approach in the context of a global health crisis.

### Limitations

This study had several limitations. The first limitation is that a major part of the interviews with the CM users and the entire topic analysis relate to the period before December 1, 2020; the influence of changes in the test policy and the app after that date could not be properly determined. The second limitation was the representativeness of this study. People with a migration background, middle and lower education levels, and those aged >70 years were underrepresented. This is pertinent because these groups are less likely to work from home [18] and run a higher risk of developing a more severe illness because of lower vaccination coverage or age [19]. Another issue related to generalizability is that critical CM users may have participated in the study. The latter is related to our enrollment procedure. A third limitation related to the sample was that all interviewees had reasonable to good digital skills. It is expected that problems identified with the app will be greater or might differ for people with more limited digital skills and people with a low educational level, mild intellectual disability, or migration background. Although the issues mentioned here negatively affect generalization, the results from the first CM evaluation study [9] and quantitative studies [6] show a picture similar to this second qualitative study. Finally, the contact-tracing study was conducted among only 3 of 25 geographically spread MHSs, which may also limit generalizability.

### Conclusions

The evaluation of the CM app with end users, designers, and the MHS provided useful recommendations for the CM CTA. The lessons learned can be used to position CTAs as digital solutions for the next global health crisis and provide insights for the development and implementation of digital solutions in general.

The CM app is a CTA that is easy to use and supports intuitive use. However, the adherence to the prescribed actions (eg, sharing a “key” in case of being infected to alert other users) is low, owing to misunderstanding of the working mechanism of the app, a design that is not based on the mental logic of the end user, problems with the accuracy of notifications in 2020, the testing policy, and the uncertainty associated with receiving notifications in general. Issues that could have, at least in part, been foreseen and proactively tackled by an intervention in the technical or communication domain. Moreover, Blasimme et al [16] showed that (national) governments and health departments are responsible for the infrastructure and education on CTAs, which strongly affects the end user’s adoption and use. For example, choices made during the build process of the app allow for the minimization of collected data and a fluent user experience through a sufficient level of infrastructure, thereby lowering barriers to use. Moreover, the choices made in the area of communication result in a level of education on CTAs that affects the user’s familiarity and trust in such techniques [20]. In these areas, this study shows shortcomings in the approach of the (national) government and where future eHealth initiatives can improve.

The added value of the CM app is tracing risks at an earlier time than traditional contact tracing and through this, increasing the chances of breaking the chain of infections. To realize this, CM should complement or relieve traditional contact tracing. However, this potential was not realized because the interaction between the infected individual and the MHS was problematic and slow, and embedment of the CM app in MHS processes was too severely limited.

The adoption of the CM app was low. In particular, the use of the app among older adults and younger people is low [6]. The CM app was prototyped by those with lower digital literacy skills. However, the communication campaigns did not motivate younger people to use the app [21]. The incentives to download the app were based on solidarity and vulnerability to protect others, such as the older adults, which now seem to be less effective among the young.

Lack of leadership and fragmentation in governance (between the Dutch Ministry of Health, Welfare and Sport, MHS, and local government) caused distrust in the COVID-19 measurements. Besides, there was no attention by the Dutch Ministry of Health, Welfare and Sport in press conferences to the added value of the CM app, for example, to use the app as an instrument that facilitates reopening society when people increasingly come into contact with others (van’t Klooster et al [17]).

Overall, this study is a testament to how a coherent strategy and process in the design, implementation, and embedment of eHealth apps, especially digital CTAs, can contribute to pandemic preparedness. To that end, Multimedia Appendix 6 [14,16,19,22,23] contains a proposal about how the lessons of this study can contribute to a strategy of that increases pandemic preparedness. Moreover, the mirroring and action approach used in this study and the focus on distilling actionable improvements to both the procedure and app provides a template for a mechanism through which future eHealth apps can rapidly be evaluated and improved even during a crisis.

## Appendix

Multimedia Appendix 1 I. Application to Ethical commission.

Multimedia Appendix 2 II. Survey questions (Dutch).

Multimedia Appendix 3 III. Interview questions for the interviews with CM app users.

Multimedia Appendix 4 IV. Interview questions for the interviews with MHS employees.

Multimedia Appendix 5 V. Original quotes and their translations.

Multimedia Appendix 6 VI. Measures for preparedness of future pandemics.

## Abbreviations

CM: CoronaMelder
CTA: contact-tracing app
MHS: Municipal Health Service
