# Addendum: The Dutch COVID-19 Notification App: Lessons Learned From a Mixed Methods Evaluation Among End Users and Contact-Tracing Employees

**DOI:** 10.2196/44497

**Published:** 2022-12-02

**Authors:** Joris Elmar van Gend, Jan Willem Jaap Roderick van 't Klooster, Catherine Adriana Wilhelmina Bolman, Julia Elisabeth Wilhelmina Cornelia van Gemert-Pijnen

**Affiliations:** 1 The BMS Lab Faculty of Behavioural, Management and Social Sciences University of Twente Enschede Netherlands; 2 Department of Psychology Open University of the Netherlands Heerlen Netherlands; 3 Section of Psychology Health & Technology, Department of Technology, Human and Institutional Behavior Faculty of Behavioural, Management and Social Sciences University of Twente Enschede Netherlands

In “The Dutch COVID-19 Notification App: Lessons Learned From a Mixed Methods Evaluation Among End Users and Contact-Tracing Employees” (JMIR Form Res 2022;6(11):e38904), the authors made the following changes:

1. An *Acknowledgments* section was not included in the initial manuscript and the authors desire this to be added to the published article. The following statement has been added under the new *Acknowledgments* section:

The authors would like to thank the following groups and individuals for their contribution to the research project. First, Prof Dr Wolfgang Ebbers in his role as head of the research team  CoronaMelder. Second, the researchers would like to acknowledge all the anonymous participants from the end users and MHS (“GGD”) employee groups. Lastly, the team would like to thank those employees of the Dutch MHS communication and CM app development teams that helped reflect on and refine the findings and the local newspaper that helped with the recruitment of participants.

2. The incorrect city was added to affiliation 2. The original affiliation was:

^2^Department of Psychology, Open University of the Netherlands, Utrecht, Netherlands

The corrected version is:

^2^Department of Psychology, Open University of the Netherlands, Heerlen, Netherlands

The correction will appear in the online version of the paper on the JMIR Publications website on December 2, 2022, together with the publication of this correction notice. Because this was made after submission to PubMed, PubMed Central, and other full-text repositories, the corrected article has also been resubmitted to those repositories.

