# Supplementary material for: The Dutch COVID-19 Notification App: Lessons Learned From a Mixed Methods Evaluation Among End Users and Contact-Tracing Employees
Source: JMIR Form Res. 2022 Nov 4;6(11):e38904. doi: 10.2196/38904 (PMC9640195; doi:10.2196/38904)
Supplement: Multimedia Appendix 1 [file formative_v6i11e38904_app1.docx]

## Application to Ethical commission

201323 REQUEST FOR ETHICAL REVIEW

#### FACULTY BMS

Request nr: 201323

Researcher: Witte, E.R. de

Supervisor: Gemert-Pijnen, J.E.W.C. van

Reviewer: Klooster, P.M. ten

Status: Approved by commission

Version: 2

1. START

A. TITLE AND CONTEXT OF THE RESEARCH PROJECT

1. What is the title of the research project? (max. 100 characters)

Contract research Opdracht kwalitatief onderzoek CoronaMelder notificatie app VWS

1. In which context will you conduct this research?

Academic research conducted by a faculty member

1. Date of the application

05-11-2020

1. Is this research project closely connected to a research project previously assessed by the BMS Ethics Committee?

Yes

please provide the ethic request number(s) for the research project(s):

200953

B. CONTACT INFORMATION

1. Contact information for the lead researcher 6a. Initials:

#### E.R.

6b. Surname:

de Witte

6c. Education/Department (if applicable):

#### EEMCS-BSS

6d. Staff or Student number:

76685098

6e. Email address:

[e.r.dewitte@utwente.nl](mailto:e.r.dewitte@utwente.nl)

6f. Telephone number (during the research project):

+31657918191

6g. If additional researchers (students and/or staff) will be involved in carrying out this research, please name them:

Prof. dr. J van Gemert-Pijnen (UT), j.vangemert- pijnen@utwente.nl; Dr. ir. J. van ’t Klooster (UT), j.vantklooster@utwente.nl; Prof. dr. C. Bolman (OU), catherine.bolman@ou.nl; M. Schreijer (UT), m.a.schreijer@student.utwente.nl; J. van Gend (UT), j.e.vangend@utwente.nl;

6h. Have you completed a PhD degree?

No

1. Contact information for the BMS Supervisor 7a. Initials:

#### J.E.W.C.

7b. Surname:

van Gemert - Pijnen

7c. Department:

#### BMS-PGT

7d. Email address:

[j.vangemert-pijnen@utwente.nl](mailto:j.vangemert-pijnen@utwente.nl)

7e. Telephone number (during the research project):

+31534896050

1. Is one of the ethics committee reviewers involved in your research? Note: not everyone is a reviewer.

No

C. RESEARCH PROJECT DESCRIPTION

9a. Please provide a brief description (150 words max.) of the background and aim(s) of your research project in non-expert language.

An in-depth study focused on the behaviour of the CoronaMelder app (CM) users after receiving a notification. Goal is to gain insight in extent to which the CM-app contributes to intended and unintended effects, especially in target groups that are hard to reach. The study specifically studies adherence to the given advice (by the CM app) in a risk situation (when receiving a notification, when infected and when having symptoms) and the contribution of these actions to contact tracing. 1. Determining adherence behaviour, persistence of CM use, and underlying motivations and limiting / promoting factors of CM

users who received a notification. 2. Determining how the BCOer (contract tracing employees) interacts with index (positively tested app user), which obstacles and difficulties the BCOer experiences, and how to reduce them. 3. Finding out the questions GP’s receive after CM users receive a notification and which possibilities they see for reducing the possible additional burden it may cause.

9b. Approximate starting date/end date of data collection:

Starting date: 2020-12-06

End date: 2021-01-30

9c. If applicable: indicate which external organization(s) has/have commissioned and/or provided funding for your research.

Commissioning organization(s):

VWS (Dutch Ministry of Health)

Funding organization(s):

#### VWS

Grant number:

BMSLAB2020.05.11 Offerte VWS kwalitatief Onderzoek naar Ervaringen met de CoronaMelder app

2. TYPE OF STUDY

Please select the type of study you plan to conduct:

I will be collecting new data from individuals acting as respondents, interviewees, participants or informants.

4. RESEARCH INVOLVING THE COLLECTION OF NEW DATA

A: RESEARCH POPULATION

1. Please provide a brief description of the intended research population(s):

CM-users, BCO-staff members and general practitioners.

1. How many individuals will be involved in your research?

CM-users: N=50 interviews.. Respondents will be recruited via schools, Pharos, low literacy institutes, blue collar companies etc). The will also be recruited via a short survey in Panelclix in which they will be asked to do an interview. BCOers and GPs: N=16 interviews (8 per profession)

1. Which characteristics must participants/sources possess in order to be included in your research?

CM users: 1) who received a notification, and 2) who used CM to notify close contacts after a positive test. We focus on hard-to-reach groups such as people with low education, limited reading and digital skills, a migration background, and the elderly. They are underexposed in existing research on the use and adherence to the CM notification. BCO employees and GPs: need to be familiar with the CM

1. Does this research specifically target minors (<16 years), people with cognitive impairments, people under institutional care (e.g. hospitals, nursing homes, prisons), specific ethnic groups, people in another country or any other special group that may be more vulnerable than the general population?

No

1. Are you planning to recruit participants for your research through the BMS test subject pool, SONA

No

B. METHODS OF DATA COLLECTION

1. What is the best description of your research?
   - (Online) survey research
   - Interview research
2. Please describe the activities that participants in your research will perform, max. 2000 characters, including spaces:

CM users (survey + interviews): Respondents are approached through various channels. Via panel research at Panelclix, respondents are generated based on selection criteria (low level of education). They then complete the survey. If they indicate that they have received a CM notification; key ,they will be asked to participate in the interview (online or face-to-face). People are also asked to participate in an interview through the following channels; schools (secondary, ROC), GGD, participants in lab tests and ethical user research, Pharos, Stichting Lezen en Schrijven and Stichting ABC, Blue collar companies, telephone helpdesk of the CM and football clubs Twente (via visitor survey)). If they want to participate in an interview, an appointment is scheduled and the interview is then conducted. Note; we take the corona measures into account, we will not visit respondents that are in quarantine or that suffer from any symptoms that might be related to COVID-19. We will conduct the interviews online via Microsoft Teams in that case. BCOers and GPs (interviews): BCOers from various GGDs (regional public health authorities) as well as general practitioners are asked to participate in an interview. If they want to participate, an appointment is scheduled and the interview is then conducted.

How much time will each participant spend (mention the number of sessions/meetings in which they will participate and the time per session/meeting)?

Survey: ± 10 mins Interview: ± 60 mins

C: BURDEN AND RISKS OF PARTICIPATION

1. Please provide a brief description of these burdens and/or risks and how you plan to minimize them:

The burden participants face is that they will have to invest some of their time when filling in the questionnaire or participating in an interview. It will be emphasized that participation is voluntary, anonymous and that subjects can withdraw at any time. In case the

respondent suffers from any symptoms that might be related to COVID- 19, the interview takes place online. Also, participation should not hinder their recovery. Respondents are always free to join / not join the research. Also see question 30. In case the interview is conducted face-to-face, there is a risk for exposure to COVID-19. We will do everything we can to minimize this risk, by using certified medical PPE and by following the advice/guidelines set by the government. The labprotocols for human-related research are approved by the Executive board.

1. Can the participants benefit from the research and/or their participation in any way?

Yes

Please Explain:

They might benefit from the feeling of “being heard” and based on their experiences the app and or the communication/support to help users will be further optimized. Participants will receive a small reward for their participation (VWS gratification).

1. Will the study expose the researcher to any risks (e.g. when collecting data in potentially dangerous environments or through dangerous activities, when dealing with sensitive or distressing topics, or when working in a setting that may pose ‘lone worker’ risks)?

Yes

Please Explain:

Yes, in case the interviews are conducted face-to-face, there is a risk for exposure to COVID-19. We will do everything we can to minimize this risk, by using certified medical PPE and by following the advice/guidelines set by the government.

D. INFORMED CONSENT

1. Will you inform potential research participants (and/or their legal repsentative(s), in case of non- competent participants) about the aims, activities, burdens and risks of the research before they decide whether to take part in the research?

Yes

Briefly clarify how:

Respondents will be fully informed about the purpose and the content of the research before they decide whether or not to provide consent.

1. How will you obtain the voluntary, informed consent of the research participants (or their legal repsentatives in case of non-competent participants)?

Signed

1. Will you clearly inform research participants that they can withdraw from the research at any time without explanation/justification?

Yes

1. Are the research participants somehow dependent on or in a subordinate position to the researcher(s)

(e.g. students or relatives)?

No

1. Will participants receive any rewards, incentives or payments for participating in the research?
   - Voucher, monetary value: € 50,-
2. In the interest of transparency, it is a good practice to inform participants about what will happen after their participation is completed. How will you inform participants about what will happen after their participation is concluded?
   - Participants will receive oral/written information about what the researcher(s) will do with the collected data.

E. CONFIDENTIALITY AND ANONYMITY

1. Does the dataset contain personal identifiable information that can be traced back to specific individuals/organizations?

No

1. Will you make use of audio or video recording?

Yes

- - What steps have you taken to ensure safe audio/video data storage?

Data will be anonymized (if neccessary) and will be safely stored on the secured drive of the UT.

- - At what point in the research will tapes/digital recordings/files be destroyed?

When the research is fully completed.

5. DATA MANAGEMENT

- - - I have read the UT Data policy.
    - I am aware of my responsibilities for the proper handling of data, regarding working with personal data, storage of data, sharing and presentation/publication of data.

6. OTHER POTENTIAL ETHICAL ISSUES/CONFLICTS OF INTEREST

1. Do you anticipate any other ethical issues/conflicts of interest in your research project that have not been previously noted in this application? Please state any issues and explain how you propose to deal with them. Additionally, if known indicate the purpose your results have (i.e. the results are used for e.g. policy, management, strategic or societal purposes).

The results will be used for a report for the Realisation and adoption team VWS, for the Communication plan about the CM app and for the house of representatives ( Tweede Kamer). The results will also be used for follow-up tests (monitoring use app 1 and app 2) and will be available for VWS.

7. ATTACHMENTS

PIF participanten_kwalitatiefOZ_coronamelder upload.pdf

8. COMMENTS

Klooster, P.M. ten ( 06-11-2020 10:46):

NB: The approval given for your research project is CONDITIONAL. As your study intends to make use of methods requiring social and physical interaction, this poses risks for both participants and researchers, which have to be taken into account. You have to COMPLY with the current RESTRICTIONS ON SOCIAL AND PHYSICAL INTERACTION

regarding the COVID19 outbreak. This may imply that you have to find alternative ways to collect data or to delay the start of your study until the restrictions have been adjusted or lifted. If adjustments lead to substantive changes in the design of your study (excluded: digital/online means to get in contact with your participants), send your changes to [ethicscommittee-bms@utwente.nl](mailto:ethicscommittee-bms@utwente.nl) stating your request number. Please consult the standing guidelines of the UT and national authorities on research and educational activities [www.utwente.nl/corona](http://www.utwente.nl/corona)

9. CONCLUSION

Status: Approved by commission

The ethical committee has assessed the ethical aspects of your research project. On the basis of the information you provided, the committee does not have any ethical concerns regarding this research project. It is your responsibility to ensure that the research is carried out in line with the information provided in the application you submitted for ethical review. If you make changes to the proposal that affect the approach to research on humans, you must resubmit the changed project or grant agreement to the ethical committee with these changes highlighted.

Moreover, novel ethical issues may emerge while carrying out your research. It is important that you re- consider and discuss the ethical aspects and implications of your research regularly, and that you proceed as a responsible scientist.

Finally, your research is subject to regulations such as the EU General Data Protection Regulation (GDPR), the Code of Conduct for the use of personal data in Scientific Research by VSNU (the Association of Universities in the Netherlands), further codes of conduct that are applicable in your field, and the obligation to report a security incident (data breach or otherwise) at the UT.
