# Supplementary material for: The Dutch COVID-19 Notification App: Lessons Learned From a Mixed Methods Evaluation Among End Users and Contact-Tracing Employees
Source: JMIR Form Res. 2022 Nov 4;6(11):e38904. doi: 10.2196/38904 (PMC9640195; doi:10.2196/38904)
Supplement: Multimedia Appendix 2 [file formative_v6i11e38904_app2.docx]

**Q1.1 Beste heer/mevrouw, Zoals u misschien weet, heeft het Ministerie van Volksgezondheid, Welzijn en Sport (VWS) op 10 oktober een app gelanceerd in de strijd tegen het coronavirus, de CoronaMelder (afgekort CM). Over dit onderzoek In opdracht van VWS voeren de Universiteit Twente en de Open Universiteit onderzoek uit naar het gebruik van de CoronaMelder. Het doel is na te gaan welke reacties en acties de CoronaMelder teweeg brengt, na een melding (dat u in de buurt bent geweest bij iemand besmet met corona) en na een positieve testuitslag (geven mensen de sleutel door?). Door de panelclix vragenlijst en het interview hopen de onderzoekers zicht te krijgen op de ervaringen met de CoronaMelder na een melding en/of na besmet te zijn met corona. De inzichten worden gebruikt om de CoronaMelder zelf, de ondersteuning daaromheen (bijv. helpdesk) en de communicatie over de corona maatregelen via de CoronaMelder te verbeteren. Uw hulp daarbij is van groot belang. We vragen u eerst om de vragenlijst in te vullen. Dat kost u hoogstens 5 minuten. Als de onderzoekers van de Universiteit Twente en de Open universiteit u daarna willen benaderen voor een interview vraagt Panelclix u eerst of u dit wilt. Als u aangeeft dat te willen, wordt u door hen benaderd. Als u meedoet aan het interview krijgt u een tegoedbon van 50€. We kunnen het interview online uitvoeren. Als dit voor u niet mogelijk is kunnen we het interview ook bij u thuis doen of in onze coronaproof interviewbus. Dit onderzoek is beoordeeld en goedgekeurd door de “BMS ethische commissie” van de Universiteit Twente. Voor eventuele vragen, opmerkingen of klachten over dit onderzoek kunt u een mail sturen naar onderzoek_coronamelder@ou.nl. Toestemming Door ‘Ja’ aan te vinken geeft u aan bovenstaande informatie te hebben gelezen en gaat u akkoord met uw deelname aan het onderzoek. U geeft de onderzoekers toestemming om uw antwoorden uit dit vragenlijstonderzoek te gebruiken voor wetenschappelijk, beleidsrelevant en maatschappelijk relevant onderzoek. Uw gegevens worden daarbij anoniem behandeld.**

**Q1.2 Geef hieronder aan wat voor u van toepassing is.**

o Ja, ik geef toestemming. Ik vul deze vragenlijst in. (1)

o Nee, ik geef geen toestemming. Ik vul deze vragenlijst niet in. (2)

**Q2.1 Om de CoronaMelder te kunnen gebruiken, moet u deze eerst installeren. Dat kan voor een iPhone via de App Store. Voor een Android smartphone kan dat via Google Play. Als u de CoronaMelder heeft geïnstalleerd, kunt u de CoronaMelder openen. Welke situatie is op u van toepassing?**

o Ik heb de CoronaMelder geïnstalleerd. (1)

o Ik heb de CoronaMelder in het verleden geïnstalleerd, maar op dit moment niet meer. (2)

o Ik heb de CoronaMelder nooit geïnstalleerd. (3)

**Q3.1 De CoronaMelder stuurt een melding als u minstens 15 minuten dicht bij iemand bent geweest die later corona blijkt te hebben (zie afbeelding). Het kan dan zijn dat u daardoor ook besmet bent geraakt. De melding verschijnt dan op uw smartphone.**

**Hoe vaak heeft u een melding gehad?**

o Een keer. (1)

o Meerdere keren. (2)

o Geen enkele keer. (3)

Q3.2

Display This Question:

If De CoronaMelder stuurt een melding als u minstens 15 minuten dicht bij iemand bent geweest die la... = Een keer.

Or De CoronaMelder stuurt een melding als u minstens 15 minuten dicht bij iemand bent geweest die la... = Meerdere keren.

**Q3.3 Wanneer heeft u deze melding gehad? Als u meerdere meldingen heeft gehad, geef dan de datum en tijd van de laatste melding. Gebruik het volgende format Dag(01)/Maand(03)/Jaar(2020).**

Datum:

________________________________________________________________

Display This Question:

If De CoronaMelder stuurt een melding als u minstens 15 minuten dicht bij iemand bent geweest die la... = Een keer.

Or De CoronaMelder stuurt een melding als u minstens 15 minuten dicht bij iemand bent geweest die la... = Meerdere keren.

**Q3.4 Tijd:**

________________________________________________________________

**Q4.1 Bent u besmet geweest met het coronavirus? Heeft u vervolgens wel of niet de sleutel doorgegeven aan de GGD medewerker? Selecteer de optie die voor u van toepassing is. Het doorgeven van de sleutel is stap 1.**

**Dit ziet er anders uit na 1 december. Afhankelijk van de datum van de melding, heeft u een van beide afbeeldingen gezien: Afbeelding van vóór 1 december (zie hieronder aan de linkerzijde) Afbeelding van na 1 december (zie hieronder aan de rechterzijde)**

o Ja, ik ben besmet geweest met het coronavirus. Ik heb de sleutel **wél** doorgegeven aan de GGD-medewerker. (1)

o Ja, ik ben besmet geweest met het coronavirus. Ik heb de sleutel **niet** doorgegeven aan de GGD-medewerker. (2)

o Nee, ik ben niet besmet geweest met het coronavirus. (3)

o Wil ik liever niet zeggen. (4)

Q4.2

**Q4.3 Linker afbeelding is een voorbeeld van de situatie voor 1 december en de rechter afbeelding van na 1 december.**

Display This Question:

If Bent u besmet geweest met het coronavirus? Heeft u vervolgens wel of niet de sleutel doorgegeven... = Ja, ik ben besmet geweest met het coronavirus. Ik heb de sleutel <strong>wél </strong>doorgegeven aan de GGD-medewerker.

**Q4.4 Heeft u na het doorgeven van de sleutel anderen gewaarschuwd door via de CoronaMelder een melding te versturen? (met anderen worden CoronaMelder app gebruikers bedoeld) Het versturen van de melding is stap 3. Dit ziet er anders uit na 1 december. Afhankelijk van de datum van de melding, heeft u een van beide afbeeldingen gezien: Afbeelding van vóór 1 december (zie hieronder aan de linkerzijde) Afbeelding van na 1 december (zie hieronder aan de rechterzijde)**

o Ja (1)

o Nee (2)

Display This Question:

If Bent u besmet geweest met het coronavirus? Heeft u vervolgens wel of niet de sleutel doorgegeven... = Ja, ik ben besmet geweest met het coronavirus. Ik heb de sleutel <strong>wél </strong>doorgegeven aan de GGD-medewerker.

**Q4.5**

**Q4.6 Linker afbeelding is een voorbeeld van de situatie voor 1 december en de rechter afbeelding van na 1 december.**

**Q5.1 Wat is uw leeftijd?**

________________________________________________________________

**Q5.2 Wat is uw geslacht?**

o Man (1)

o Vrouw (2)

o Anders (3)

**Q5.3 Wat is de hoogste opleiding die u heeft afgerond?**

o Lagere school/basisonderwijs (1)

o Lager Algemeen of Lager Beroeps Onderwijs (lbo, lts, leao, en dergelijke) (2)

o Middelbaar Algemeen Onderwijs ((m)ulo, mavo, mms) (3)

o Middelbaar Beroeps Onderwijs (mbo, mts, meao) (4)

o Hoger Algemeen Onderwijs (havo, hbs, vwo) (5)

o Hoger Beroeps Onderwijs (hts, heao, hbo, pabo, en dergelijke) (6)

o Wetenschappelijk onderwijs (universiteit) (7)

Display This Question:

If De CoronaMelder stuurt een melding als u minstens 15 minuten dicht bij iemand bent geweest die la... = Een keer.

Or De CoronaMelder stuurt een melding als u minstens 15 minuten dicht bij iemand bent geweest die la... = Meerdere keren.

Or Bent u besmet geweest met het coronavirus? Heeft u vervolgens wel of niet de sleutel doorgegeven... = Ja, ik ben besmet geweest met het coronavirus. Ik heb de sleutel <strong>wél </strong>doorgegeven aan de GGD-medewerker.

Or Bent u besmet geweest met het coronavirus? Heeft u vervolgens wel of niet de sleutel doorgegeven... = Ja, ik ben besmet geweest met het coronavirus. Ik heb de sleutel <strong>niet</strong> doorgegeven aan de GGD-medewerker.

**Q5.4 Wilt u deelnemen aan een interview over uw ervaringen met de CoronaMelder app? Het interview duurt 45 minuten tot een uur. We kunnen het interview online uitvoeren. Als dit voor u niet mogelijk is kunnen we het interview ook bij u thuis doen of in onze coronaproof interviewbus. Als u meedoet aan het interview krijt u een tegoedbon van 50€.**

o Ja (1)

o Nee (2)
