# Supplementary material for: The Dutch COVID-19 Notification App: Lessons Learned From a Mixed Methods Evaluation Among End Users and Contact-Tracing Employees
Source: JMIR Form Res. 2022 Nov 4;6(11):e38904. doi: 10.2196/38904 (PMC9640195; doi:10.2196/38904)
Supplement: Multimedia Appendix 3 [file formative_v6i11e38904_app3.docx]

**1. Hoe lang gebruikt u de app al? (lancering = 10 oktober; pretest periode aug-okt),**

1. Welke telefoon (IOS/Android)?

Scenarios benoemen:

1. **Melding** (dicht bij iemand geweest die later besmet blijkt te zijn met corona)
2. **Test aanvragen**
3. **Sleutel doorgeven GGD**
4. **Zelf melding versturen om anderen te waarschuwen**
5. **Blijf Thuis/ISOLATIE**

De CoronaMelder stuurt een melding als u minstens 15 minuten dicht bij iemand bent geweest die later besmet bleek te zijn met het coronavirus. De melding verschijnt dan op uw smartphone.

**2. Heeft u zo'n melding van de Corona Melder ontvangen? (screenshots invoegen/voor en na 1 december)**

1. Kunt u aangeven wanneer, op welk moment en hoe vaak (datum en locatie) u die ontvangen heeft?
2. Had u lichamelijke klachten die leken op de symptomen van corona (aan het begin , of paar dagen later? of had u geen klachten?

**3. Wat was uw eerste reactie toen u de melding ontving, wat voelde u, wat dacht u, wat deed u?** (bijv. bezorgd/angstig, boos, onverschillig, in de war)

**4. Wat vond u van de inhoud van de melding?** (onduidelijk, duidelijk, begrijpelijke boodschap etc.)

1. kunt u dat aangeven, bijv op screenshot (a/b afhankelijk van meld datum)
2. in hoeverre was voor u duidelijk wat u moest doen? waarom wel / niet (vervolgvragen komen later (vraag 6)
3. Had u het idee dat de melding klopte of niet? doorvragen: kunt u aangeven wat en waarom u dat dacht? A

**5. Wat deed u, direct nadat u de melding ontving?** (bijv. melding bewaard op telefoon, foto gemaakt, opgeschreven, hulp gevraagd, app verwijderd, meteen HA/GGD gebeld?)

**6. Wat deed u daarna?** Respondent laten vertellen wat hij/zij heeft gedaan.

**7. advies scenario na melding (voor en na 1 dec/screenshots invoegen)**

De app kan verschillende adviezen geven in een gestuurde melding. We gaan de adviezen 1 voor 1 doornemen.

1. **Blijf thuis tien dagen nadat u misschien besmet bent geraakt.** (thuis isolatie)
2. Herkent u dit advies uit de app?
3. Wat heeft u toen gedaan? (bijv. wel of niet thuisgebleven) Waarom?
4. Wat vindt u van dit advies? Wat zou anders moeten en waarom?
5. Was het voor u uitvoerbaar, waarom wel/niet?  (welke hindernissen, welke stimulans heeft u ervaren) **Voorbeelden laten noemen.**

1. **Laat anderen boodschappen doen.**
   1. Herkent u dit advies uit de app?
   2. Wat heeft u daar mee gedaan? Waarom?
   3. Wat vindt u van dit advies? Wat zou anders moeten volgens u en waarom?
   4. Was het voor u uitvoerbaar, waarom wel / waarom niet (welke hindernissen, welke stimulans heeft u ervaren) **Voorbeelden laten noemen.**

1. **Alleen mensen die bij je wonen, mogen samen met jou in huis zijn. Blijf bij elkaar uit de buurt en houd 1,5 meter afstand. Dus niet knuffelen, niet zoenen en geen sex.**
   1. Herkent u dit advies uit de app?
   2. Wat heeft u daar mee gedaan? Waarom?
   3. Wat vindt u van dit advies? Wat zou anders moeten volgens u en waarom?
   4. Was het voor u uitvoerbaar, waarom wel / waarom niet (welke hindernissen, welke stimulans heeft u ervaren) **Voorbeelden laten noemen.**

1. **Ontvang geen bezoek.**
   1. Herkent u dit advies uit de app?
   2. Wat heeft u daar mee gedaan? Waarom?
   3. Wat vindt u van dit advies? Wat zou anders moeten volgens u en waarom?
   4. Was het voor u uitvoerbaar, waarom wel / waarom niet (welke hindernissen, welke stimulans heeft u ervaren) **Voorbeelden laten noemen.**

1. **Medische hulp nodig? Raadpleeg eerst de huisarts.**
   1. Herkent u dit advies uit de app?
   2. Wat heeft u daar mee gedaan? Waarom?
   3. Wat vindt u van dit advies? Wat zou anders moeten volgens u en waarom?
   4. Was het voor u uitvoerbaar, waarom wel / waarom niet (welke hindernissen, welke stimulans heeft u ervaren) **Voorbeelden laten noemen.**

1. **Heb je (lichte) klachten die passen bij het coronavirus? Doe een coronatest en blijf tien dagen thuis.**
   1. Herkent u dit advies uit de app?
   2. Wat heeft u daar mee gedaan? Waarom?
   3. Wat vindt u van dit advies? Wat zou anders moeten volgens u en waarom?
   4. Was het voor u uitvoerbaar, waarom wel / waarom niet (welke hindernissen, welke stimulans heeft u ervaren) **Voorbeelden laten noemen.**
   5. In geval van test gedaan:
      1. op wat voor manier? (bijv. via de CoronaMelder app, coronatest.nl, testnummer (0800-1202) of huisarts) , na 1 dec is **er een ander meld nummer en screenshot , dus die ook invoegen**


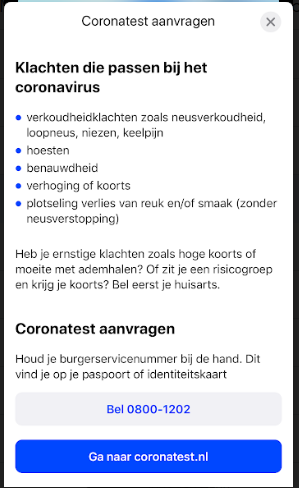


1. Waarom op die manier?
2. Bij welke teststraat getest? (bijv GGD, commercieel)
   1. Waarom op die manier?
3. Wanneer test aangevraagd? (bijv. direct naar melding, of dagen later) en wanneer gedaan?

1. Was het voor u uitvoerbaar, waarom/waarom niet (welke hindernissen, welke stimulans heeft u ervaren, voorbeelden laten noemen

**8. In hoeverre had u het idee dat u al deze adviezen moest uitvoeren?** Waarom wel / niet en  wat vind u hiervan? Wat zou het makkelijker maken om de adviezen op te volgen?

**9. Welke acties heeft u nog meer ondernomen?** (bijv. app verwijderd, werkgever op de hoogte gesteld); en waarom?

**10. scenario test aanvragen: screenshot corona test aanvragen**

Heeft u een corona test aangevraagd ?

Zo ja, wat was de reden dat u zich heeft laten testen? (was dit vanwege de app melding? Of had dit een andere reden?)

1. hoe heeft u de test aangevraagd? (bij. website coronatest.nl, testnummer 0800-1202, via de Coronamelder app of via uw huisarts) let op **VOOR en NA 1 dec anders dus beide screenshots laten zien**
   1. Waarom op die manier?
2. wat voor soort test heeft u gedaan? (GGD test; sneltest/commerciële test/welke; zelftest), en waar en wanneer)? (**voor en na 1 dec andere opties, dus uitvragen nav datum van test**)
   1. Waarom?
3. Was het voor u uitvoerbaar om een test aan te vragen, waarom/waarom niet (welke hindernissen, welke stimulans heeft u ervaren, voorbeelden laten noemen
4. hoe hebt u de testuitslag tot u genomen (via digiD zelf online opzoeken, gebeld, beide)
5. Wat kan er volgens u nog verbeterd worden?

**11.  scenario sleutel doorgeven**

***screenshots***

Bent u positief getest op een moment? zo ja, hebt u toen de sleutel gedeeld, waarom wel of niet. wat vond u moeilijk / makkelijk. Hoe zou u het anders willen zien? - wat zou het makkelijker voor u maken? en dan 2 stappen: sleutel delen met GGD en sleutel delen in app vragen. Als u de uitslag zelf online hebt opgezocht, hebt u er dan nog aan gedacht om het via de app door te geven? Is u dit gevraagd door de GGD medewerker die u belde? (komt later ook terug), misschien combineren

*
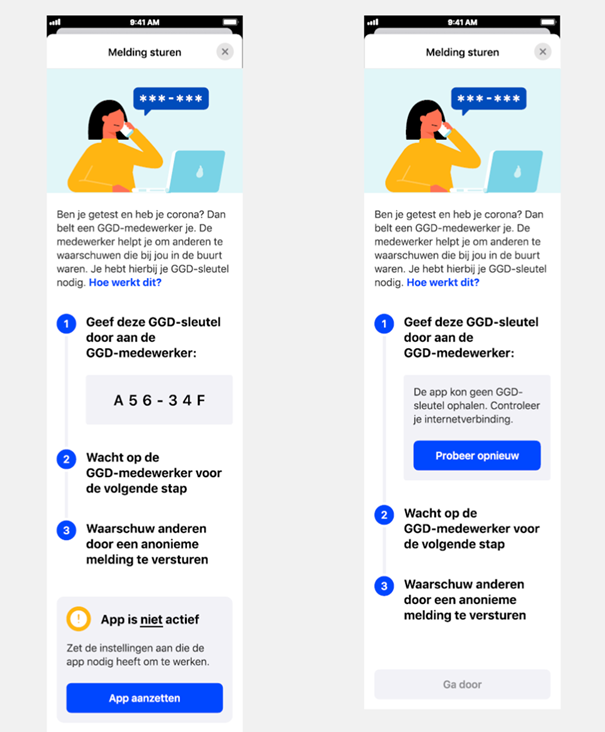
*

**12. scenario sleutel opzoeken in app**

screenshots erbij nemen van voor en na 1 dec

**ahv screenshots vragen stellen over 3 stappen, zie app**

**
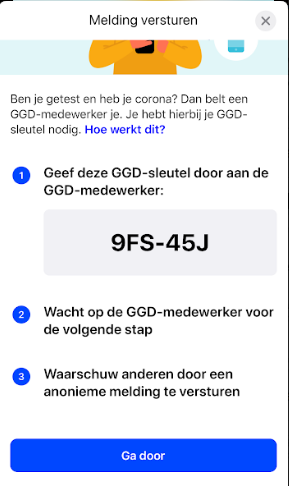
**

deze slide laat zien als u een sleutel ontvangt wat u dan kan doen

1. Kon u de sleutel vinden? Wist u waar u moest zoeken in de app? Kon u dat tegelijkertijd met het telefoneren met de ggd medewerker?
2. wat heeft u daarmee gedaan? foto gemaakt, gebeld, bewaard , hoe
3. was het voor u uitvoerbaar, waarom/waarom niet (welke hindernissen, welke stimulans heeft u ervaren, voorbeelden laten noemen
4. Wat zijn verbeterpunten volgens u?

**13. Scenario sleutel en GGD contact (screenshots)**

1. heeft de GGD u benaderd? wanneer en hoe? wat vond u daarvan?  heeft u toen de ‘sleutel’ doorgegeven aan de GGD medewerker?
2. Hoe ging dat? Wat was uw ervaring daarmee? Zie reacties ook bij vragen melding.
3. wat heeft u toen vervolgens gedaan en waarom? (app verwijderd, bellen , niks etc, door vragen vanuit respondent zijn antwoord)

      b. was het voor u uitvoerbaar, waarom/waarom niet (welke hindernissen, welke      stimulans heeft u ervaren, voorbeelden laten noemen.

1. Wat zijn verbeterpunten volgens u?

**14. scenario: sleutel doorgeven in app om een melding te sturen om andere CM gebruikers te waarschuwen (screenshots)**

**hier vragen aan de hand van de 3 stappen stellen, zie screenshots per stap vragen opnemen wat gedaan, hoe ervaren, welke hindernissen etc**

1. Stap 1: heeft u de sleutel ook doorgegeven: S**CREENSHOTS ERBIJ NEMEN**

**
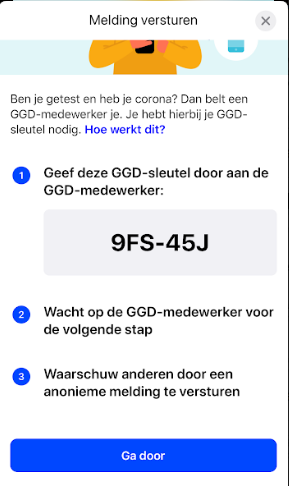
**

1. Stap 2: wacht op ggd medewerkers> vragen hierover stellen
2. Stap 3: waarschuw andere CM gebruikers door een anonieme melding te versturen

i. hoe verliep dat? heeft u daarbij hulp gehad/gevraagd etc

ii. heeft u een reactie ontvangen op het sleutel doorgeven, in de app?

iii. Zo nee, waarom niet? (bijv. wist ik niet, lukte mij niet, ik wilde het niet)

1. was het voor u uitvoerbaar, waarom/waarom niet (welke hindernissen, welke stimulans heeft u ervaren, voorbeelden laten noemen
2. Wat zijn verbeterpunten volgens u?

**15. scenario: isolatie na besmet te zijn (screenshots) en dat van voor en na 1 dec, want de isolatie is verkort**

1. hoe heeft u dat ervaren, wat heeft u gedaan? heeft u hulp gevraagd etc etc
2. Welke acties heeft heeft u nog meer ondernomen toen u met corona besmet was? (bijv. in quarantaine, werkgever ingelicht, contact opgenomen met bijv. huisarts/GGD, CM app verwijderd), waarom, welke hulp had u nodig ? etc
3. was het voor u uitvoerbaar, waarom/waarom niet (welke hindernissen, welke stimulans heeft u ervaren, voorbeelden laten noemen
4. wat zijn verbeterpunten volgens u?

**16. vragen over houding deelnemer richting CoronaMelder**

1. Wat is uw houding tegenover de CoronaMelder?/Hoe staat u tegenover de CM?
2. In hoeverre bent u van mening dat de CoronaMelder helpt bij het verminderen van het aantal coronabesmettingen? Kunt u dat toelichten?
3. Heeft u aanvullende verbeterpunten (die u hiervoor niet heeft genoemd)?
4. Wat zijn dingen die u goed vindt aan de app? Positieve punten?

**Deel 2: Achtergrondinformatie deelnemer**

**Vraag 17**

Wat is uw leeftijd?

**Vraag 18**

Wat is uw hoogst afgeronde opleidingsniveau? (bijv. basisonderwijs, vmbo, mbo1 of havo onderbouw, mbo 2-4)

**Vraag 19**

(Health literacy, chew 2004, ook in Liss panel gebruikt)

Hoe vaak helpt iemand u met het lezen van brieven of folders van uw huisarts, het ziekenhuis of andere zorginstellingen?

**Vraag 20**

In welke sector bent u werkzaam/welke functie heeft u?

**Vraag 21**

U gebruikt de app, en mogelijk andere digitale communicatiemiddelen, maar hoe zou u uw digitale vaardigheden omschrijven, bijvoorbeeld als u uzelf een cijfer van 1-10 zou geven? (1 is heel slecht, 10 is heel goed)

**Vraag 22**

Uit welke provincie en stad of dorp komt u (momenteel woonachtig)?

**Vraag 23**

Welke mobiele telefoon gebruikt u? (Welk besturingssysteem?)
