# Supplementary material for: The Dutch COVID-19 Notification App: Lessons Learned From a Mixed Methods Evaluation Among End Users and Contact-Tracing Employees
Source: JMIR Form Res. 2022 Nov 4;6(11):e38904. doi: 10.2196/38904 (PMC9640195; doi:10.2196/38904)
Supplement: Multimedia Appendix 4 [file formative_v6i11e38904_app4.docx]

**1. Algemeen**

1. Hoe lang werk je al als BCO’er?
2. Hoeveel uur werk je per werkdag? en per werkweek?
   1. Hoeveel indexen spreek je (gemiddeld) per dag?
3. Hoeveel tijd kost het om één index te behandelen?
4. Heb je de training gevolgd?  → Training BCO en training CM zijn losse trainingen, beide uitvragen
   1. Waarom wel / niet?
5. Wat vond je  van de training (on-the-job en e-learning) voor gebruik CM app in BCO?
   (Training BCO-ers gaat over sleutel delen CM app; Registratie testuitslagen na een                melding via CM app)
6. Heb je het gevoel dat je voldoende bent ingewerkt om het BCO onderzoek te doen?
   1. In het bijzonder de CM-gerelateerde handelingen (dus sleutel doorgeven en melding versturen)?
7. Wat kan anders, beter aan trainingen/opleidingen? Welke problemen of hindernissen ben je hierbij tegengekomen? Hoe was dat bij collega BCO’ers?
8. Doe je het werk als BCO’er voor 1 GGD of voor meerdere GGD’en?
   1. Zo ja; in hoeverre is de procedure hetzelfde?
   2. Bij welke GGD werk je?
9. Wat is jouw persoonlijke houding tegenover de CM app als BCO’er?
10. In hoeverre denk je dat de CM app helpt bij het verminderen van het aantal Corona besmettingen?

**2. Acties na een melding via de CM app**

• **Acties na een positieve testuitslag**

1. Welke protocollen gebruik je bij een positieve uitslag?
2. Welke registratie verricht wanneer je contact hebt met de index?
   1. In welke systemen van GGD?
   2. Welke vragen stel je daarbij zoal?
   3. Vraag je of de index de CM app gebruikt?
   4. Vraag je of de index een melding heeft gekregen?
   5. Welke extra vragen stel je wanneer er een melding via CM app aan vooraf ging?
   6. Welke extra acties voer je uit wanneer de index de CM app gebruikt?
3. Hoe worden testuitslagen (positief en negatief) gecommuniceerd naar geteste persoon? (en in hoeverre maakt het dan uit of men de CM gebruikt (welk kanaal)
4. Van alle indexen die u spreekt, hoeveel % (schatting) heeft ongeveer de app?
5. Wordt de GGD contact app toegepast binnen het BCO proces?
   1. Zo ja, hoe? En hoe bevalt dit?
6. Wat voor problemen ervaar je hierbij (wat betreft CM onderdeel)? Hoe ga je daarmee om?
7. In hoeverre is dit landelijk geregeld of alleen voor jullie afdeling zo geregeld?

• **Hoe verloopt de procedure van sleutel delen?**

1. Hoe wordt het contact met de index georganiseerd?
   1. Wat is meestal het tijdsbestek (dus tijd tussen uitslag en BCO (en dus ook sleutel doorgeven))?
   2. Kan het tijdsbestek variëren? Met welke reden?
2. Attendeer je de index op het belang van sleutel delen en hoe dit moet?
   1. Probeer je dit in dezelfde sessie te doen?
   2. Hoe wordt de sleutel geregistreerd?
   3. Hoe wordt index bijgestaan in sleutel delen?
   4. Komt het weleens voor dat de index de sleutel niet wil delen?
3. Wordt het versturen van de melding via de CM app (stap 3, dus na sleutel delen) ook begeleid door BCO’er?
   1. **Krijgt BCO bevestiging van versturen melding?**
4. Wat zijn de verdere BCO acties na sleutel delen contact?
5. Wat kan beter, anders? welke problemen ervaar je hierbij (dus het contact met index wat betreft het doorgeven van sleutel en versturen van melding via CM app)?

**3. Thuisisolatie**

1. Merk je verschillen in indexen met en zonder app als het gaat om thuisisolatie (bijv. beter geïnformeerd)?
   1. Zo ja; welke?
   2. En waarom is dat denk je?
2. Informeer je indexen over thuisisolatie? zo ja welke info geef je (belang, hoe etc)
3. In hoeverre krijg je vragen over thuisisolatie? Waar gaan die over?
4. Hoe kan thuisisolatie beter verlopen?

**4. Verbeterpunten en conclusies**

• **Hoe kan CM app beter worden betrokken in BCO? testen. traceren, thuisisolatie?**

1. Uit interviews met CM app gebruikers komt naar voren dat de tijd tussen test uitslag en sleutel delen/contact BCO als lang ervaren wordt.
   1. Waarom kan het zo lang duren voordat het BCO wordt gedaan? Welke belemmerende en faciliterende factoren zijn er? o.a. testuitslag via DigiD
2. Hoe kan het contact met de CM-gebruiker na positieve melding geoptimaliseerd (o.a. in snelheid) worden? welke aanpassingen zijn nodig bv in het protocol voor sleutel delen ?
3. Hoe kan registratie in IT systemen (Hp zone, coronIT etc) van sleuteldelers geoptimaliseerd worden?
4. Hoe kan de CM app beter in werkproces BCO geïntegreerd worden?
   1. Wat is daar voor nodig?
5. Hoe kan het aanvragen van een test (na CM melding) beter/sneller verlopen?
6. Veel mensen bellen toch naar GGD voor een afspraak in de teststraat (ipv digitaal) omdat zij meer opties zien en je daarmee eerder een afspraak kan krijgen. Is dat zo en wat vindt u daarvan?
7. Hoe is de overgang van symptomatisch testen naar asymptomatisch testen (na 1 dec)?
   1. Ervaring mbt testaanvragen cm app?
8. Hoe werkt sleuteldeling na commerciële test?
   1. Wat gaat goed, wat minder?
9. In hoeverre is informatie in CM app eenduidig met die van GGD?
   1. Waar zitten discrepanties en waarom?
10. Wat zijn de uitdagingen voor de BCOer mbt de aansluiting van de CM (sleutel delen) en het BCO onderzoek?
    1. Oa. zorgde de werkdruk door de 2^e^ golf voor problemen?
11. Waar zit volgens jou de meeste winst in het optimaliseren van CM app tbv BCO?
12. Draagt CM app bij aan eerder en sneller waarschuwen in uw optiek? Kunt u dat onderbouwen vanuit de contactonderzoek ervaringen?

**Over BCO’er zelf (demografische info):**

1. Wat is je leeftijd?
2. Wat is je opleidingsniveau?
3. Aanvullende info??
