# Supplementary material for: The Dutch COVID-19 Notification App: Lessons Learned From a Mixed Methods Evaluation Among End Users and Contact-Tracing Employees
Source: JMIR Form Res. 2022 Nov 4;6(11):e38904. doi: 10.2196/38904 (PMC9640195; doi:10.2196/38904)
Supplement: Multimedia Appendix 5 [file formative_v6i11e38904_app5.docx]

## Original quotes and their translations

*“On Monday 31 Augustus, I read on my CoronaMelder that I had been close for more than 15 minutes to someone who reportedly was infected. It was a big shock! That, according to me, wasn’t possible! But, anyway, I called the phone number that was provided. I got the advice to go in quarantine and to call my general practitioner or the MHS if symptoms developed. I called my friends and family! The Monday afterwards I received another notification from the CM app. I had not been in contact with anyone infected according to the app. I didn’t understand this at all!”*

*“Maandag 31 augustus, zag ik op mijn corona melder dat ik in de buurt was geweest ,langer dan 15 minuten, met iemand die besmet zou zijn! Ik schrok me dood ! Dat kon volgens mij niet waar zijn! Maar enfin het opgegeven telefoonnummer gebeld. Ik kreeg het advies om in quarantaine te gaan en bij evt. klachten de huisarts of GGD te bellen. Ik heb mijn familie en vrienden gebeld! Maandag daarop kreeg ik weer een bericht op de melder. Ik was met niemand in aanraking geweest volgens de melder. Nou ik begreep er niet veel van!” -* MAIL0116

*“After reading your article in the newspaper, I would like you to know that I have removed the app [CM] from my phone and this is why: On 2 November I received a message that I had been in contact for more than 15 minutes with someone that has corona and that I had to stay inside until 30 Ocotber.”*

*Naar aanleiding van Uw artikel in de krant, wil ik u laten weten dat ik de app weer van mijn telefoon afgegooid heb en wel om de volgende reden:* *op 2 november kreeg ik een berichtje dat ik op dinsdag 20 oktober langer dan 15 minuten in contact ben geweest met iemand met corona en dat ik tot 30 oktober binnen moest blijven.”* - MAIL0072

*“My experience is that the phones of the neighbours and myself had been in contact. (...) It can happen that people get a notification [from CM] that is incorrect, but better this than the other way around.”*

*“Dus mijn ervaring is dat de mobiels van de buren en van mij contact hebben gehad. (…) Maar het kan dus zijn dat mensen een melding krijgen die niet klopt, maar ja beter zo dan andersom.”* - MAIL0074

*“After yet another sleepless night I concluded that I still did not experience any symptoms. To get rid of all the brooding, I made an appointment with the branch of [a commercial test provider] in Hengelo [town].”*

*“Na weer een slapeloze nacht constateerde ik, dat ik nog steeds geen klachten had. Om van dat gepieker af te komen, heb ik een afspraak gemaakt met de vestiging van covidtestnederland.nl in Hengelo.”* - MAIL0209

“My daughter in law owns a hairsalon in [city], which is now closed as well. She has 5 people working for her en had the CoronaMelder. She removed it. She indicated that the app drove her crazy.” - CME008 (male, 71 years old, high level of education)

“Mijn schoondochter heeft een kapsalon in *plaats*, die zit nu ook dicht, heeft 5 mensen aan het werk, had ook die CoronaMelder erop zitten die heeft hem er gewoon afgegooid. (...) Ze zegt ik word helemaal gek van dat ding zegt ze.” - CME0008 (man, 71 jaar, HBO)

“I had already received a Whatsapp message in the morning from someone whom I had exercised with that they were positive. Therefore, I already knew that I had to take action. I had a sore throat ache, but I doubted a bit. Then the CM notification came and that felt more serious.” - CME0146 (female, 58 years, high level of education)

“Ik had ’s ochtends al een appje gehad van haar van iemand waar ik dus mee gesport had. Dat zij positief was. Dus ik wist al van ik moet actie gaan ondernemen want ik had zelf wat keelpijn. Maar ik twijfelde nog een beetje en toen kwam die CoronaMelder en die vond ik eigenlijk indringender dan haar appje.” - CME0146 (vrouw, 58 jaar, HBO)

“I know that I was supposed to have had a contact on 29 October and that I received the notification on the Sunday (8 November) afterwards. This, on the moment when my quarantine period had elasped, I received the notification.” - CME0109 (female, 45 years, middle level of education)

“Ik weet wel dat ik 29 oktober dan een contact zou moeten hebben gehad en de zondag daarna (8 november), dus op het moment dat mijn quarantaine periode al afgelopen was, toen kreeg ik de melding pas.” - CME0109 (vrouw, 45 jaar, MBO)

“Because that same day I also called the MHS, but it was not possible to get through. So then I called again the next day.” - CME0104 (male, 59 years, high level of education)

“Want diezelfde dag heb ik ook gebeld met de GGD, maar daar was geen doorkomen aan. Dus toen heb ik de volgende dag weer gebeld.” - CME0104 (man, 59 jaar, HBO)

“I was scared to death. You receive a notification that you were in contact with someone who was infected with corona on the 17th of October. That made me think: Where was I on that Saturday? What was I doing? I don’t go to the market or store. I started thinking and I really thought hard about it. It gave me sleepless nights and I never figured out where I had been and who I had been in contact with.” - CME0017 (male, 65 years, Middle level of education).

“Ik ben me doodgeschrokken. En dan krijg je dus de melding dat je op 17 oktober in aanraking bent geweest met iemand die besmet is met corona. En dan ga je nadenken van: waar was ik op die zaterdag? Wat heb ik gedaan, want ik kom namelijk niet op de markt, ik kom namelijk niet in de winkel. […] En toen ben ik eens gaan nadenken en ik heb me suf gedacht en slapeloze nachten gehad en ik ben er nog tot nu toe nog nooit achter gekomen waar ik geweest ben en met wie ik in aanraking ben geweest.” - CME0017 (man, 65 jaar, MBO)

“But I did call all those people in the meantime. And those people were not affraid either, they were happy that I had called them and that was it.” - CME0008 (male, 71 years, high level of education)

“Maar ik heb wel al die mensen gebeld in tussentijd. En die mensen waren ook niet bang hoor die waren blij dat ik gebeld had en dat was het.” - CME0008 (man, 71 jaar, HBO)

“What do you do: You are going to think about where I had been and what I had done that day. And you can’t figure it out properly and you thus become irritated.” - CME0125 (female, 67 years, low level of education)

“Want wat doe je: je gaat na waar ben ik die dag allemaal geweest wat heb ik allemaal gedaan. En je komt er niet helemaal goed uit dus je wordt een beetje kriegel dat je er niet helemaal uitkomt.” - CME0125 (vrouw, 67 jaar, voortgezet onderwijs)

“Yes, but my husband and I live with just the two of us, so that’s not applicable. (...) My husband and I lie next to each other in 1 bed, so that is not going work out.” - CME0006 (female, 62 years, high level of education)

“Ja, maar mijn man en ik wonen met zijn tweeën hier, dus dat is allemaal niet van toepassing zeg maar. (...) Mijn man en ik liggen naast elkaar in 1 bed, dus dat gaat niet lukken.” - CME0006 (vrouw, 62 jaar, HBO)

“It was my daughter’s birthday when my son was infected. My daughter then sat downstairs with some friends and my son was upstairs. We didn’t say, at that point in time: No one is allowed into the house.” - CME0031 (female, 50 years, high level of education).

“Ook toen zeg maar mijn ene zoon besmet was, toen was mijn dochter jarig. Nou ja, toen heeft mijn dochter wel beneden gezeten met een paar vrienden en mijn zoon was gewoon boven. Maar we hebben toen niet gezegd er komt niemand in huis.” - CME0031 (vrouw, 50 jaar, HBO)

“At the MHS, the problem at that point in time was that their capacity was too limited for the number of requests they received. It was known at that time to be the case. That was quite a big hassle and irritator. However, it was easily arranged at a commercial test centre, so that was a good experience.” - CME0227 (male, 35 years, high level of education)

“Ja bij de GGD was de hindernis gewoon dat de capaciteit gewoon te laag was op dat moment voor het aantal aanvragen, dat is natuurlijk ook wel bekend dat dat toen gewoon was. Dus dat was een flinke hindernis en ergernis. Maar bij die commerciële partij was het zo gepiept dus dat was op zich een prima ervaring.” - CME0227 (man, 35 jaar, HBO)

“So then I was tested, but my symptoms weren’t severe enough. I also coudln’t get a test immediately. Well, that was what the girl on the phone said “You don’t have symptoms”. However, I then told her that I had been in contact with someone that had been infected and that I’d like to be tested to be sure. I said that I might have experienced mild symptoms in the throat, but I don’t think that I would have had myself tested with those symptoms under normal circumstances. I did it to make the situation more severe so I could get tested.” - SME 0042 (female 62 years, high level of education)

“Dus toen heb ik een test laten doen, maar mijn klachten waren niet echt erg. Ik kon ook niet direct een test laten doen. Of tenminste, het meisje dat ik aan de telefoon had, had zoiets van ‘ja je hebt geen klachten’. Maar toen heb ik aangegeven dat ik toch wel contact heb met iemand die besmet is (geweest), dus dat ik mij voor de zekerheid wou laten testen. Ik heb gezegd dat ik misschien wel lichte keelklachten had, maar dat was normaal gesproken denk ik niet mee naar een test gegaan. Om het een beetje aan te dikken omdat ik toch een test wilde laten doen.” - CME0042 (vrouw, 62 jaar, HBO)

“He was tested on Tuesday evening and was called on Thursday afternoon that his results were negative. In the meantime, we had drawn the conclusion that his test result would be negative, but it caused him a lot of stress in the meantime. Particularly because he knew the test result was available. But that they then waited for 1.5 days to call him, was pretty frustrating.” - CME0028 (female, 36 years, middle level of education)

“Hij is op dinsdagochtend getest, hij werd op donderdagmiddag gebeld met een uitslag dat hij negatief was. Dan hadden wij ondertussen zelf al wel de conclusie getrokken dat het negatief zou zijn, maar dat heeft bij hem voor heel veel stress gezorgd. Vooral omdat hij wist dat de uitslag er was, want hij had die bevestiging via de mail. Maar dat het dan nog anderhalve dag duurt voordat hij gebeld werd, was het vrij frustrerend.” - CME0028 (vrouw, 36 jaar, MBO)

“Because of this, you also start doubting the effectiveness of other things, like the CM app. I had assumed that the hospital would have shared the positive test result. (...) But in my case, the hospital hadn’t communicated the positive test result with the MHS.” - CME0426 (male, 69 years, education unknown)

“Daardoor ga je ook twijfelen aan de werking van allerlei dingen, bijvoorbeeld de Corona Melder app. Ik was ervan uitgegaan dat het ziekenhuis de positieve test door hadden gegeven. (...) Maar mijn voorbeeld, was toen dat het ziekenhuis de positieve test niet doorgaf aan de GGD.” - CME0426 (man, 69 jaar, opleiding onbekend)

“And that was the next day, so on Monday the 19th I had a person on the phone about contact tracing. They asked whether I had been in contact with people. I told them that I hadn’t been in contact with many people, I had been in contact with the physical therapist. They asked me if I could inform them myself and wanted to hang up. I then said that I had the CM app and asked if I could do something with it. They ten told me to provide the code, so they could report it. That I did. However, if I hadn’t told them (...) THen they would have just asked me to inform others and nothing else.” - CME0179 (male 70 years, high level of education)

“En dat was de volgende dag, dus maandag de 19e kreeg ik iemand aan de telefoon over contactonderzoek. En die zei bent u in contact geweest met mensen? Ik zeg nou niet te veel maar met de fysiotherapeut, nou wilt u die dan zelf informeren. En toen wilde ze ophangen en toen zei ik van maar ik heb ook een CoronaMelder, kan ik daar ook nog iets mee? Ja dan moet u mij de code doorgeven dan zal ik het melden. Dus dat heb ik toen gedaan. Maar als ik het niet gezegd had… (...) Dan had ze gewoon aan mij gevraagd wilt u mensen informeren en voor de rest niet.” - CME0179 (man, 70 jaar, HBO)

“No, because I assumed that the MHS would do that. (...) Because the MHS asked me for the key and it wasn’t clear to me that I had to finish the rest of the procedure.” - CME0179 (male, 70 years, high level of education)

“Nee want ik ging ervanuit dat de GGD dat zou doen. (...) Omdat de GGD vroeg mij om de sleutel en voor de rest was mij niet duidelijk dat ik dan verder nog de procedure moest afwerken.” - CME0179 (man, 70 jaar, HBO) (over melding versturen na sleutel delen)

“But if you get a notification and you stay inside, then you cannot infect anyone else and then it won’t spread as much. So yes, absolutely.” - CME0006 (female, 62 years, high level of education)

“Maar als je dus een melding krijgt en je blijft binnen, dan kun je niemand verder aansteken en dan verspreidt het zich naar mijn mening veel minder. Dus ja, absoluut.” - CME0006 (vrouw, 62 jaar, HBO)

“My brother in law, for example, received a notification as well, but afterwards it turned out that he wasn’t infected. You have to be close to someone for 15 minutes, but he says he wasn’t. So that makes you doubt a bit, whether that was good. Is it [CM app] functioning 100%?” - CME0006 (female, 62 years, high level of education)

“Mijn zwager bijvoorbeeld heeft ook zo’n melding gekregen, maar achteraf bleek die niet besmet geraakt te zijn gelukkig. En een kwartier moet je in de buurt zijn geweest bij iemand, maar hij zegt dat hij dat gewoon niet is geweest. Dus dat brengt je een beetje aan het twijfelen, of dat wel goed zou zijn. Zou die wel voor 100% werken?” - CME0006 (vrouw, 62 jaar, HBO)

“Lately, I have conversations of an hour and the time needed for administration is at least an hour as well. Sometimes even longer, depending on where someone has been. Thus, it is very hard for me to make an estimation of it [time spent per index]. Often, it takes longer than expected.”- CME1009 (female, 25 years, high level of education)

“De laatste tijd heb ik wel gesprekken van een uur en dan is de uitwerktijd ook minstens wel een uur. Soms ook langer zelfs, afhankelijk van waar diegene allemaal is geweest. Dus daar kan ik soms lastig een inschatting van maken. Vaak duurt het langer dan gedacht.” - CME1009 (vrouw, 25 jaar, WO)

“And the training is very theoretically good, and you think that this is how it will go. And then you start working, and you notice that it doesn’t go like they said in the instructions. So you do learn useful skills, but in the end those conversations are different. Mainly because they are less scary. The instruction makes it seem like everyone is constantly angry and is going to threaten your life, but I never experienced this.” - CME 1008 (male, 26 years, high level of education)

“En die training is heel theoretisch goed, en je denkt dat is hoe het gaat verlopen. En dan begin je eenmaal met werken, en dan merk je dat het eigenlijk helemaal niet zo gaat zoals ze in de werkinstructie hebben verteld. Dus je leert wel nuttige skills ervoor, maar uiteindelijk zijn de gesprekken toch net iets anders omdat ze minder eng zijn vooral. De werkinstructie doet het voordoen alsof iedereen constant boos op je gaat zijn en je leven gaat bedreigen, maar uiteindelijk heb ik dat nooit meegemaakt.” - CME1008 (man, 26 jaar, WO)

“I don’t use the [CM] app myself, because I have the idea that it gets used too little to be truly useful. I think, that if I were to be infected, that I wouldn’t be warned through the app.” - CME1008 (male, 26 years, high level of education)

“Ikzelf gebruik het niet omdat ik het idee heb dat het te weinig gebruikt wordt om daadwerkelijk nuttig te zijn. Ik denk dat als ik besmet zou raken, dat ik niet gewaarschuwd zou worden door de app.” - CME1008 (man, 26 jaar, WO)

“What we are missing, and we pointed that out yesterday, is that we need more depth. It all remains a bit superficial.” - CME1007 (female, 28 years, high level of education)

“Wat wij heel erg missen, dat hebben we gisteren ook aangegeven, is dat we meer de diepte ingaan. Het blijft een beetje aan de oppervlakte.” - CME1007 (vrouw, 28 jaar, HBO)

“I don’t use the [CM] app myself, because I have the idea that it gets used too little to be truly useful. I think, that if I were to be infected, that I wouldn’t be warned through the app.” - CME1008 (male, 26 years, high level of education)

“Ikzelf gebruik het niet omdat ik het idee heb dat het te weinig gebruikt wordt om daadwerkelijk nuttig te zijn. Ik denk dat als ik besmet zou raken, dat ik niet gewaarschuwd zou worden door de app.” - CME1008 (man, 26 jaar, WO)

“The way in which I do my contact tracing, I do not explicitly ask for it [use of CM app]. Maybe a link could be added that if you make an appointment and it is because of the CM app, that it automatically adds this in the system. When we get the test results, it then reads “This person is warned by the app.” That would be a way to make that clear. At this moment there are several ways into the test centres. You can call because you experience symptoms, you can also call because you were warned by the CM app or because you are in quarantine for 5 days. We currently do not see information about this in the system.” - CME1000 (male, 29 year, highly educated).

“Op de manier waarop ik specifiek mijn stijl van brononderzoek heb, vraag ik er dus niet per se expliciet naar. Misschien zou je een link kunnen hebben dat als je de afspraak maakt, dat je dan doorgeeft dat het vanwege de corona melder app is en dat die dan automatisch in het systeem, als wij het testresultaat binnen krijgen, erbij staat ‘deze persoon is gewaarschuwd door de app’. Dat zou een manier zijn om dat duidelijk te maken. Er zijn op het moment verschillende ingangen bij de teststraat. Je kan bellen omdat je klachten hebt, je kan ook bellen omdat je door de corona melder app gewaarschuwd bent, of omdat je in quarantaine zit voor 5 dagen. Daar zien wij op het moment nog geen informatie over in het systeem.” - CME1000 (man, 29 jaar, WO).

“The reason why they don’t share the keys is because they didn’t know that they had to provide information. Why they don’t know that, is something I don’t know.” - CME1006 (male, 44 years, high level of education)

“De reden waarom ze de sleutel niet doorgeven is omdat ze niet wisten dat ze iets moesten doorgeven en waarom dat ze dat niet weten dat weet ik niet.” - CME1006 (man, 44 jaar, HBO)
