# Supplementary material for: The Dutch COVID-19 Notification App: Lessons Learned From a Mixed Methods Evaluation Among End Users and Contact-Tracing Employees
Source: JMIR Form Res. 2022 Nov 4;6(11):e38904. doi: 10.2196/38904 (PMC9640195; doi:10.2196/38904)
Supplement: Multimedia Appendix 6 [file formative_v6i11e38904_app6.docx]

# Measures for preparedness of future pandemics

To use digital solutions for effective global epidemic surveillance, tracing apps like CoronaMelder should be reconsidered.

First, to overcome fragmentation of contact tracing apps, a vision is needed on how to use privacy by design principles with user centred design. The Corona Melder and other comparable European tracing apps demand for strong leadership and a clear (international) policy on digital solutions during a crisis, like the current pandemic. A pandemic resilience framework should be developed to position tracing apps as an element of a package of measurements instead of a standalone app. Such a framework should discuss data sharing and data transfer to support and optimise Contact Tracing procedures and interoperability between different tracing apps (international setting) [22]. Besides, a data management plan is needed to enable monitoring of the use of tracing apps. This implies a discussion about the balance between privacy by design and user centred design.

Second, incentives are important to increase the adoption and use of tracing apps. End-users expected an app that provides more technical features than just an alert or notification app. For example, features to monitor symptoms. This allows the app to serve more as a central platform for anything related to the COVID-19 pandemic with a more closed off process funnel that could in turn encourage adherence and participation.

Third, adequate, targeted communication campaigns are needed for adoption of contact tracing apps. The contact tracing apps in other European countries faced similar adoption issues and a number of downloads below expectation [16]. A lack of trust regarding government, lack of insights in the purpose of these apps, doubt about privacy may have contributed to such relatively low adoptions [16]. The national government, health departments are responsible for adequate communication, however, this requires a clear leadership about authorities during a pandemic, to communicate who rules the pandemic and to frame messages adaptive to the spread of the virus and the measurements based on it.

Last but not least, our findings indicate that the CoronaMelder app can be complementary to the traditional, manual Contact Tracing, as for example described by Ebbers [14]. This is reflected as well in the interaction of errors on the infrastructure, technical and procedural sides discussed by the participants. There errors enlarge each other´s negative outcomes, e.g.: The period most participants discuss happens to coincide with the period in which the MHS had limited capacity for COVID-19 testing and was at time not contactable due to the high amounts of queries they received [23]. Participants indicated that this not just impacted their perception of the MHS’ approach, but also the CM app. On the other hand, the CM app is able to detect high-risk contacts between its users earlier and notify them faster. To optimise this, users should be able to activate the exposure code (the key) without contacting the Contact Tracing-professionals. Recently this has been made possible after an update to the app and underlying procedures, resulting in higher reported numbers of codes being shared with the MHS [19].
